# The Rise of Allogeneic Natural Killer Cells As a Platform for Cancer Immunotherapy: Recent Innovations and Future Developments

**DOI:** 10.3389/fimmu.2017.00631

**Published:** 2017-05-31

**Authors:** John P. Veluchamy, Nina Kok, Hans J. van der Vliet, Henk M. W. Verheul, Tanja D. de Gruijl, Jan Spanholtz

**Affiliations:** ^1^Department of Medical Oncology, VU University Medical Center, Cancer Center Amsterdam, Amsterdam, Netherlands; ^2^Glycostem Therapeutics, Oss, Netherlands

**Keywords:** hematopoietic stem cell transplantation, autologous natural killer cells, allogeneic natural killer cells, adoptive natural killer cell therapy, natural killer cell biotech companies, natural killer cell combinatorial studies

## Abstract

Natural killer (NK) cells are critical immune effector cells in the fight against cancer. As NK cells in cancer patients are highly dysfunctional and reduced in number, adoptive transfer of large numbers of cytolytic NK cells and their potential to induce relevant antitumor responses are widely explored in cancer immunotherapy. Early studies from autologous NK cells have failed to demonstrate significant clinical benefit. In this review, the clinical benefits of adoptively transferred allogeneic NK cells in a transplant and non-transplant setting are compared and discussed in the context of relevant NK cell platforms that are being developed and optimized by various biotech industries with a special focus on augmenting NK cell functions.

## Natural Killer (NK) Cells in Oncology

So far T cells have been the mainstay of cancer immunotherapy; however, it is generally recognized that NK cells also play an essential role in antitumor immunity. Certainly in the prevention of metastases through the elimination of circulating cancer stem cells with a high metastatic potential, NK cells are recognized as main immune effector cells (1). Moreover, as solid tumors have a propensity to particularly down-regulate MHC-I, NK cells provide a failsafe mechanism in these circumstances where cytotoxic T cells, which depend on MHC-I for tumor recognition and elimination, are debilitated. NK cells have recently been more intensely explored as a viable therapeutic platform next to T cell-based approaches. This review aims to summarize the latest developments in the clinical translation of adoptive transfer of NK cells in the oncology field.

## NK Cells and Their Activating and Inhibitory Receptors

Human NK cells are generally categorized by their level of CD56 and CD16 expression into two subsets: CD56^bright^CD16^dim^ and CD56^dim^CD16^bright^ NK cells. Most NK cells in the peripheral blood and spleen are CD56^dim^CD16^bright^ and are cytotoxic against a variety of tumor cells, whereas CD56^bright^CD16^dim^ NK cells are immune regulatory in function and constitute the majority in secondary lymphoid tissues, producing abundant cytokines but exerting weak cytotoxicity compared to CD56^dim^CD16^bright^ NK cells (2). The ability of NK cells to discriminate between a cancer cell and a healthy cell is regulated by a balance between its activating and inhibitory receptors. NK-activating receptors such as DNAM-1 and NKG2D; natural cytotoxicity receptors (NCRs) such as NKp30, NKp44, NKp46, CD94/NKG2C, CD94/NKG2E, and CD16a; and activating killer cell-immunoglobulin like receptors (KIRs) contribute to NK cell activation, triggering the release of cytotoxic granules and proinflammatory cytokines such as interferon gamma (IFNγ) from NK cells to lyse cancer cells (3). The NK cell-activating receptor NKG2D (CD314) recognizes MHC class-I-chain related proteins A and B (MICA and MICB) and ULBPs (1–6), while DNAM-1 binds to CD112 (Nectin-2) and CD155 (poliovirus receptor) (5) on stressed, infected, and cancer cells. The ligands for NCRs are widely expressed on cells infected by viruses or by intracellular bacteria and on tumor cells, but their exact modes of action are yet to be characterized to define their role in NK cytotoxicity (6). In addition to this, the heterodimers of the NKG2 family; CD94/NKG2C and CD94/NKG2E recognize the non-classical MHC class I molecule HLA-E and associate with DAP-12 molecule to trigger an NK activation signal (7, 8). Another very important activation mechanism of NK cells is through the interaction of CD16a (FcγRIIIa, a low affinity Fc receptor) with the Fc portion of IgG_1_ antibodies, forming an immunological synapse to engage antibody opsonized targets for NK cell-mediated antibody-dependent cell mediated cytotoxicity (ADCC) (9). Besides engaging activating receptors, NK cells also induce target cell death using tumor necrosis factor α (TNF-α), Fas ligand, and TNF-related apoptosis-inducing ligand (TRAIL) (10). The most prominent NK cell inhibitory receptors include inhibitory KIRs that recognize MHC class I (HLA-ABC) molecules, which are universally expressed on healthy tissues. Similarly, CD94/NKG2A, an inhibitory receptor from the NKG2 family, binds to HLA-E and induces NK cell tolerance through the activation of an intracellular immunoreceptor tyrosine-based inhibitory motif (ITIM) (8). Hence, knowing that NK cell functions are determined by an array of receptors, which can either potentiate an activating or inhibitory signal, depending on different ligand interactions with tumor cells, it is critical to shift the balance in a therapeutic setting toward an activating NK phenotype to expedite enhanced NK tumor killing mechanisms.

## NK Cell Dysfunctionality in Cancer

Natural killer cells can control circulating tumor cells and prevent formation of tumor metastases (11). However, tumors employ different strategies to evade killing by NK cells. Upregulation of inhibitory ligands such as MHC class I molecules (HLA-ABC, HLA-G and HLA-E) has been associated with a stronger inhibitory signal to NK cells (12–15). Furthermore, increased expression of the inhibitory NKG2A receptor reported in renal cell carcinoma resulted in decreased functionality of tumor infiltrating NK cells (16). On the other hand, downregulation of NK-activating ligands for NKG2D such as MICA and MICB and increased shedding of tumor-derived soluble MIC also impair NKG2D-mediated NK cell tumor recognition (17). Another important necessity for optimal NK cell function is the ability to home and migrate to tumor sites. Several studies have correlated increased homing of NK cells to tumor tissues with improved treatment outcomes in solid tumors (18–22). However, the immunosuppressive tumor stroma comprising regulatory T cells (T-regs) (23), myeloid-derived suppressor cells (MDSCs) (24), M2 macrophages (25), and immature dendritic cells severely restricts NK cell functionality and their entry into solid tumors. In chronic diseases, such as those associated with human immunodeficiency virus and cytomegalovirus infections, mainly exhausted NK cells with decreased cytokine production and reduced cytolytic activity are observed (26, 27). In a study with breast cancer patients, the NK cell expression levels of activating receptors (NKG2D, DNAM, CD16, and NKp30) were decreased, whereas inhibitory receptor (NKG2A) expression levels were increased and this apparent dysfunctionality of NK cells was found to directly affect NK cell cytotoxicity (28). Similarly, the effector subset of NK cells (CD56^dim^CD16^+^) from head and neck and breast cancer patients, when tested *in vitro*, was highly prone to apoptosis, thus pointing to low NK cell activity in these patients (29). Impaired NK cell functionality may result from tumor-imposed suppressive mechanisms and presents a major hurdle for NK cell-targeted immunotherapies. Therefore, approaches to restore or replace impaired NK cell cytotoxicity may prove essential for an effective host defense against cancers.

## NK Cells in the Clinic

Novel NK cell-based immunotherapeutic strategies are being developed to overcome the functional limitations of the use of cancer patients’ autologous NK cells. To increase the number of functional NK cells even in case of a high tumor load, adoptive transfer of autologous NK cells served as a very feasible approach, as this ruled out the need for immunosuppression, HLA-matching, and prevented the risk of graft versus host disease (GvHD). These advantages sparked the initiation of large-scale expansion protocols and clinical trials using autologous NK cells as a treatment modality for cancer. Though adoptive transfer of autologous NK cells resulted in an increased number of circulating NK cells in peripheral blood, it failed to produce significant therapeutic effects in hematological malignancies, metastatic melanoma, and renal cell carcinoma patients due to the inhibition by self-HLA molecules (30–32). Moreover, the expansion efficiency and functional status of autologous NK cells were still limited when compared to allogeneic NK cells, as autologous cells were often obtained from heavily pretreated patients (33). In addition to this, it was difficult to track infused autologous NK cells in patients and to study their antitumor effects from peripheral blood analyses due to the inability to differentiate *ex vivo* manipulated and transferred autologous NK cells from the non-manipulated circulating NK cells. These limitations motivated researchers shifting their focus to allogeneic NK cells to treat cancer.

In patients with leukemia undergoing allogeneic hematopoietic stem cell transplantation (HSCT), NK cells, being the first lymphoid subset to appear after allogeneic HSCT (34), play a crucial role in controlling host defense against infections and residual cancer cells before T cells are reconstituted (35). These donor T cells are prime mediators of GvHD (36), and the life-threatening complications that arise due to GvHD have completely overshadowed the beneficial effects of alloreactive NK and T cells, fueling efforts to use T cell depleted grafts (37). Further, this led to the development of NK cell-based therapies coupled with T cell depleted HSCs to enhance the graft versus tumor effect (GvT) without causing GvHD. Unlike autologous NK cells, allogeneic NK cells are not restricted by the patient’s tumor’s HLA expression, which is an added advantage to mount an improved anti-tumor effect (38, 39). Current translational efforts that are explored as anticancer therapies include adoptive transfer of *ex vivo* activated and/or expanded allogeneic NK cells, either alone or in combination with HSCT.

## Sources of Allogeneic NK Cells Used in the Clinic

Commonly used allogeneic NK cells are apheresis products collected from haploidentical and unrelated donor PBMC (40). Another source is umbilical cord blood (UCB), where NK cells are generated from CD34+ progenitor cells that undergo expansion and differentiation using cytokines and growth factors and thereby mature into cytolytic NK cells (41). Apart from PBMC and UCB, NK cells have also been obtained from the clonal cell line NK-92, derived from immortalized lymphoma NK cells (42, 43).

## Allogeneic NK Cell Therapy in a Transplant Setting

Autologous or allogeneic HSCT serves as a curative regimen by reconstituting the immune system in hematological malignancies. At an earlier stage post HSCT, NK and T cells developing from the graft are immature and less in number with reduced functionality. Under those circumstances, the infusion of purified allogeneic NK cells was explored as a viable option to target minimal residual disease (MRD), prevent graft failure, and relapse. Grafts for allogeneic HSCT and allogeneic NK cell treatments were obtained from HLA matched/mismatched and related/unrelated donors (38, 39). Earlier clinical trials performed by Passweg et al. (44), Koehl et al. (45), Shi et al. (46), Yoon et al. (47), Rizzieri et al. (48), and Brehm et al. (49) have shown that NK cells can be safely administered prior to or post HSCT in patients with different types of hematological diseases. Immune suppression is a prerequisite prior to most of the allogeneic HSCT and NK-cell infusions. A non-myeloablative conditioning regimen usually consisting of cyclophosphamide (Cy) and fludarabine (Flu) was found to facilitate NK cell persistence and expansion *in vivo* (50). High doses of Cy/Flu caused pancytopenia and resulted in high plasma IL-15 levels, which also correlated with the detection of adoptively transferred NK cells up to 14 days after infusion, thus suggesting that excess IL-15 was probably utilized by the NK cells to proliferate and persist longer *in vivo* (51). A summary of clinical trials with allogeneic NK-cell infusions in a HSCT setting with published data is summarized in Table 1, and selected clinical trials from recent years are reviewed below.

**Table 1:** **Summary of allogeneic NK cell clinical trials in a transplantation setting**.

Study	Malignancy	Clinical trial design	Culture method^a^	Infused dose NK cells	Final product characteristics	Outcome
Phase I (NCT01729091) Shah et al. (59)	MM (*n* = 12)	Conditioning with Mel on day 7 and Lnd from days 8 to 2 prior to UCB-NK-cell infusion (day 5), followed by autologous-HSCT on day 0	*Ex vivo* expanded MNCs from unrelated UCB donors. Culture duration: 14 days with irradiated K562 clone 9.mbIL-21 aAPCs and IL-2 ^a^CD3 depleted (on day 7)	Four escalating doses: 5 × 10^6^, 1 × 10^7^, 5 × 10^7^, and 1 × 10^8^ cells/kg	Mean purity: 98.9% CD56+/CD3− cells	Well tolerated. No GvHD. 4/12 progressed or relapsed (median of 21 months follow-up)
Phase I (NCT01795378) Choi et al. (58)	AML (*n* = 45) and ALL (*n* = 6)	Haplo-HSCT followed by DNKI from the same donor on days 6, 9, 13, and 20 post HSCT	*Ex vivo* expanded and activated PBNK cells from haploidentical donors. Culture duration: 2–3 weeks with IL-15 and IL-21	Four escalating doses: median DNKIs are 5 × 10^7^, 5 × 10^7^, 1 × 10^8^, and 2 × 10^8^ cells/kg	Median viability: 80%. Purity: 48–98% CD56+ CD122+ cells. 0–22% CD3+ CD56+ cells. 0–10.4% CD3+ CD56− cells	Toxicity observed in 73% of patients, 9/45 aGvHD. 29/51 CR (9.3–34.7 months follow-up), 35/51 PD
Phase I (NCT00402558) Phase II (NCT01390402) Lee et al. (57)	AML (*n* = 8), MDS (*n* = 6), and CML (*n* = 7)	Conditioning with Flu/Bu prior to haplo-allo NK-cell infusion, followed by IL-2 therapy (5×, daily); conditioning with Thy/Tac prior to HLA-matched related unrelated allo-HSCT	*Ex vivo* expanded and activated PBNK cells from haploidentical donors. Culture duration: o/n with IL-2. ^a^CD3 depleted and CD56 selected (in three infusions)	Four escalating doses: 1 × 10^6^, 5 × 10^6^, 3 × 10^7^, and 3 × 10^7^ cells/kg in Phase I study. Four escalating doses of 5 × 10^6^ cells/kg in Phase II study	Median purity: 0.02% CD3+ cells. 11.41% CD14+ cells. 21.84% CD19+ cells. 14.1% CD56+ CD3− cells	Well tolerated, no GvHD. 5/21 CR, 5/21 died of transplantation related issues and 11/21 died of relapse
Phase I (NCT01287104) Shah et al. (56)	EWS (*n* = 5), DSRCT (*n* = 3), RMS (*n* = 1)	HLA matched haplo- or unrelated allo-HSCT followed by aNK-DLI from the same donor on day 7 and 35 post HSCT	*Ex vivo* expanded and activated PBNK cells from haploidentical donors. Culture duration: 9–11 days with KT64.4-BBL artificial antigen presenting cells. ^a^CD3 depleted and CD56 selected	Repeated doses (2× doses 1, 2, and 3): 1 × 10^5^ cells/kg (dose 1), 1 × 10^6^ cells/kg (dose 2), and 1 × 10^7^ cells/kg (dose 3)	Median purity: CD3+ cells 1.4 × 10^4^ cells/kg. CD56+ cells ≥90%. Viability: ≥70%	5/9 aGvHD. 2/9 SD, 7/9 CR. 4/9 are still alive (12.5–27.4 months after treatment)
Phase I/II (NCT01220544) Killig et al. (55)	AML (*n* = 24)	Haplo-HSCT followed by NK-cell infusion from same donor and OKT3 treatment from days −5 to +3	PBNK cells from haploidentical donors. ^a^CD3 depleted and CD56 selected	Single dose: 1.61–32.2 × 10^6^ CD56+/CD3− cells/kg	Purity: CD56+ CD3− cells 99.97%. CD3+ cells 0.95–7.4 × 10^4^ cells/kg	Toxicity correlated with haplo-HSCT. Deaths: 2/24 GvHD, 6/24 infections and 7/24 died of relapse. 9/24 CR (0.1–8.6-year follow-up)
Phase I/II (NCT00823524) Choi et al. (54)	AML (*n* = 32), ALL (*n* = 7), MDS (*n* = 1), DLBCL (*n* = 1)	HLA haplo-HSCT followed by DNKI from the same donor, 14 days and 21 days after HSCT	*Ex vivo* expanded and activated PBNK cells from haploidentical donor. Culture duration: 13–20 days with IL-15, IL-21, and hydrocortisone	Escalating doses (2×): 0.2 × 10^8^ cells/kg (3 pts), 0.5 × 10^8^ cells/kg (3 pts), 1.0 × 10^8^ cells/kg (8 pts), and ≥1.0 × 10^8^ cells/kg (27 pts)	Viability: 71–85%. Median purity: CD56+ CD122+ cells >90%. CD3+ CD56+ cells <3%. Fold expansion: 0.8–70 (after 13–20 days of culture)	Well tolerated. 9/41 aGvHD, 10/41 cGVHD. In total, 11 patients died of TRM. In AML (21/29) (4/8) ALL/lymphoma are in CR
Phase I (IND # 12971) Klingemann et al. (53)	NHL (*n* = 6), MM (*n* = 5), and HL (*n* = 2)	MHC-mismatched haploidentical NK-MC infusion, 49–191 days post auto-HSCT	*Ex vivo* expanded and activated PBNK cells from haploidentical donors. Culture duration: o/n with IL-2	4 Escalating doses: 1 × 10^5^, 1 × 10^6^, 1 × 10^7^, and 2 × 10^7^ MC/kg	Median purity: 26% CD56+ CD3− cells. 0.15% CD3+ cells. Median viability: 95% post wash	Well tolerated. No GvHD. 6/13 relapsed and 7/13 in remission
Phase II (NCT01386619) Stern et al. (52)	AML (n = 8), ALL (*n* = 5), HL (*n* = 2) sarcoma (*n* = 1)	Haplo-HSCT followed by NK-DLI from the same donor, +day 3, +day 40, and +day 100 post HSCT	PBNK cells from haploidentical donors. ^a^CD3 depleted and CD56 selected	Repeated doses (2–3): 0.3–3.8 × 10^7^ cells/kg	Median purity: CD3+ cells 0.03 × 10^5^ cells/kg. Median viability: 84%	Safe and feasible. 4/16 aGvHD. Median follow-up of 5.8 years 4/16 are alive. 3/16 died from graft failure
Phase I/II (NCT01386619) Brehm et al. (49)	AML (*n* = 6), ALL (*n* = 5), NB (*n* = 5), RMS (*n* = 1) HL (*n* = 1)	Haplo-HSCT followed by IL-2 stimulated NK-cell infusion (cryo) or unstimulated NK-cell infusion (fresh) from the same donor, +da 3, +day 40, and +day 100 post HSCT	*Ex vivo* expanded and activated PBNK cells from haploidentical donors. Culture duration: 9–14 days with (group II) or without (group I) IL-2 (fresh or cryo). ^a^CD3 depleted and CD56 selected	Repeated doses (1–3 doses): Group I: 3.2–38.3 × 10^6^ cells/kg Group II: 6.0–45.1 × 10^6^ cells/kg	Purity: CD56+ CD3− cells 84.4–98.6%. CD3+ cells group I: 0.4–53.4 × 10^3^ cells/kg. CD3+ cells group II: 7.7–98.3 × 10^3^ cells/kg. Viability: freshly NK-cell unstimulated median 93%. Cryo NK-cell IL-2 stim 30–70%	Well tolerated without GvHD >grade II. Group I: 5/9 died (126–498 days post SCT), 3/9 CR (742–2,218 days). Group II: 5/9 died (27–373), 2/9 CR, and 2/9 in remission
Phase I (NCT00586690) Rizzieri et al. (48)	Lymphoma (*n* = 30)	3–6/6 HLA-matched haploidentical NK-cell infusion, –8 weeks post haplo-HSCT from the same donor	PBNK cells from haploidentical donors. ^a^Only CD56 selected	Repeated dose (1–3): median dose in 3–5/6 HLA match: 9.21 × 10^6^ CD3+/CD56− cells/kg, median dose 6/6 HLA match: 10.6 × 10^6^ CD3+/CD56− cells/kg	6/6 HLA-matched: Purity: 87–100% CD56+ cells. 0.53 ± 1.1 × 10^6^ cells/kg CD3+ CD56−. 3–5/6 HLA-matched: Purity: 86–100% CD56+ cells. 0.27 ± 0.78 × 10^6^ cells/kg CD3+ CD56−	Safe. Low toxicity. 6/6 HLA-matched: 6/14 aGvHD (1 severe) and median OS 12 months. 3–5/6 HLA-matched: 8/16 aGvHD and median OS 27 months
Phase I (NCT00569283) Yoon et al. (47)	AML (*n* = 12) MDS (*n* = 2)	HLA-mismatched HSCT followed by allo NK-cell infusion from the same donor	*Ex vivo* expanded, differentiated and activated CD34+ progenitor cells (PB-derived) from haploidentical donors. Culture duration: 21 days with FLt3, IL-7 and hydrocortisone followed by 21 days with IL-15, IL-21 and hydrocortisone	Single dose: 0.33–24.5 × 10^6^ cells/kg	Mean purity: CD56+ CD122+ cells 64%. CD3+ cells 1.0%. Mean viability: 88%	1/14 aGvHD and 4/14 cGvHD. 9/14 died (between 1.7 and 15.5 months), 4/14 CR (between 16.2 and 21.6 months) 1/14 PD (25.9 months)
(BB-IND-11347) Shi et al. (46)	MM (*n* = 10)	Conditioning with Flu/Dex/Mel followed by haplo-KIR-ligand-mismatched NK-cell infusion on day 0 and day +2; IL-2 therapy daily (11×) starting on day +1 after NK-cell infusion; auto-HSCT on day +14	*Ex vivo* expanded and activated PBNK cells from haploidentical donors. Culture duration: o/n with IL-2 (pts 1–5) and brief incubation with IL-2 and anti-CD3 beads (pts 5–10)	Combined dose (day 0 and day +2): 2.7–92 × 10^6^ cells/kg	Purity: median CD3+ cells 5.5 × 10^4^ cells/kg. Viability: 95%	Safe and no GvHD. 5/10 CR, 1/10 PR, 1/10 MR, 1/10 SD, and 2/10 PD. 4/10 are alive at 1.4, 1.5, 2.3, and 3 years post NK-cell therapy
Pilot study Koehl et al. (45)	AML (*n* = 1) ALL (*n* = 2)	Haplo-HSCT followed by KIR-mismatched NK-cell infusion on day +1 or +2 post HSCT and additional infusions every 4–6 weeks; IL-2 therapy +2 days post HSCT, every second day for 2–4 weeks	*Ex vivo* expanded and activated PBNK cells from haploidentical donors. Culture duration: >12 days with IL-2. ^a^CD3 depleted and CD56 selected (in three infusions)	Repeated doses (1–3): 8.9–29.5 × 10^6^ cells/kg (first infusion), 3.3 and 11.1 × 10^6^ cells/kg (second infusion), 33.8 × 10^6^ cells/kg (third infusion)	Purity: CD56+ CD3− cells 95%. Median CD3+ cells 0.04%, 45–1,100 × 10^3^ cells. Viability: 95%. Fold expansion: median 5 (after 14 days of culture)	Well tolerated, no GvHD. 1/3 CR (152 days), 2/3 died (80 days and 45 days after NK-cell infusion)
Passweg et al. (44)	AML (*n* = 4), CML (*n* = 1)	Haplo-HSCT followed by NK-DLI from the same donor 3–12 months post HSCT	PBNK cells from haploidentical donors. ^a^CD3 depleted and CD56 selected	Single dose: 0.21–1.41 × 10^7^ cells/kg	Median purity: CD56+ CD3− cells 97.3%. T-cell 0.22 × 10^5^ cells/kg	Well tolerated and feasible. 4/5 continuous remission (8–18 months), 1/5 PD

*CNS, central nervous system; MPDs, myeloproliferative disorders; LPD, lymphoproliferative disorder; MM, multiple myeloma; MDS, myelodysplastic syndromes; MDN, myelodysplastic neoplasms; MPN, myeloproliferative neoplasms; AML, acute myeloid leukemia; LBLL, lymphoblastic leukemia-lymphoma; ALL, acute lymphoblastic leukemia; NB, neuroblastoma; RMS, rhabdomyosarcoma; CML, chronic myelogenous leukemia; NHL, non-Hodgkin’s lymphoma; MCL, mantle cell lymphoma; DLBCL, diffuse large B cell lymphoma; FL, follicular lymphoma; ALCL, anaplastic large cell lymphoma; HL, Hodgkin’s lymphoma; RCC, renal cell cancer; SCLC, small cell lung cancer; CLL, chronic lymphocytic leukemia; HCC, hepatocellular carcinoma; PNET, primitive neuroectodermal tumor; ACC, adrenal cortical carcinoma; EWS, Ewing sarcoma; DSRCT. desmoplastic small round cell tumor; Cy, cyclophosphamide; Flu, fludarabine; Bor, bortezomib; Dex, dexamethasone; Clo, clofarabine; Eto, etoposide; Cis, cisplatin; Pac, paclitaxel; Doc, docetaxel; Vin, vinorelbine; Gem, gemcitabine; Car, carboplatin; Pem, pemetrexed; TBI, total body irradiation; Tac, tacrolimus; Pred, prednisolone; mPred, methylprednisolone; Thy, thymoglobulin; Vin, vincristine; Adr, adriamycin; Predn, prednisone; Mel, melphalan; OKT3, muromonab-CD3; HSPC, human stem and progenitor cells; aGvHD, acute graft versus host disease; DNKI, donor NK-cell infusion; Stim, stimulated; Unstim, unstimulated; DFS, disease-free survival, CR, complete remission; PR, partial response; MR, minimal response; SD, stable disease; PD, progressive disease; aNK-DLI, donor-derived IL-15/4-1BBL activated NK-cell infusion; TRM, transplant-related mortality; MC, mononuclear cell; TLS, tumor lysis syndrome; PLS, passenger lymphocyte syndrome; NE, not evaluable*.

*^a^Culture method displays CD3 depleted PBMC’s, otherwise deviated selection method is mentioned*.

In 2013, Stern et al. treated acute myeloid leukemia (AML), Acute Lymphocytic Leukemia, Hodgkin’s lymphoma (HL), and sarcoma patients with allogeneic NK cells (CD3 depleted and CD56 selected) after a haploidentical HSCT, using the same donor as NK cell source. An overall survival (OS) of 25% was achieved during a median follow-up of 5.8 years. And 4/16 patients developed acute GvHD (aGvHD) due to high T-cell impurities present in two NK cell products and two from stem cell grafts, both containing ≥0.5 × 10^5^ cells/kg T cells (52). Although this prospective Phase II study reported the safety and feasibility of NK-cell infusion following allo-HSCT, it failed to yield results in support of anti-leukemia effects, raising questions as to whether the NK cell dosage of 0.3–3.8 × 10^7^ cells/kg used was sufficient to induce a clinical effect. In the same year, Klingemann et al. published data highlighting the safety and alloreactivity of HLA-mismatched (CD3 depleted) NK cells, transfused after autologous HSCT in multiple myeloma (MM), non-Hodgkin’s lymphoma (NHL), and HL patients. In this study, 13 patients were enrolled; 6/13 relapsed and 7/13 were in remission during a follow-up between 144 and 1,158 days following autologous stem cell transplantation. The allogeneic NK cells were well tolerated without GvHD. In addition, this study also demonstrated that NK cells generated and processed at distant centers can be shipped and transfused without significantly affecting the viability and cytotoxicity of the NK cell product (53).

Choi et al. (54) summarized their observations from a study, in which allogeneic *ex vivo* expanded and activated NK cells derived from the same donor were administered 14 and 21 days post HLA haploidentical HSCT to patients with hematological malignancies (*n* = 41). The data set from this study was compared with a group of 31 patients, who underwent only HLA haploidentical HSCT. A significantly higher progression-free survival (PFS) was seen in the HSCT + NK group compared to HSCT only group (74% versus 46%). In addition to this, the occurrence of chronic GvHD (cGvHD) (15% versus 10%) and transplant-related mortality (27% versus 19%) was reduced in the HSCT + NK group compared to the HSCT only group (54). In another study by Killig et al. (55), AML patients were treated with haploidentical HSCT followed by NK-cell infusions (CD3 depleted and CD56 selected) from the same donor, on days +1 and +2 post HSCT. aGvHD was highly prevalent in 20/24 patients in this study and histological analysis of skin revealed that GvHD was associated with infiltration by perforin^+^CD8^+^ T cells. Allogeneic NK cells contributed to an increased OS in the HSCT + NK group compared to the HSCT only group (37% versus 14%) over a median follow-up of 2 years (55).

Subsequently, Shah et al. published data from a Phase I study treating patients with Ewing sarcoma, rhabdomyosarcoma, and desmoplastic small round cell tumors (*n* = 9), using donor-derived IL-15/4-1BBL-activated allogeneic NK cells (CD3 depleted and CD56 selected) following allogeneic HSCT from the same donor. aGvHD was highly prevalent in the patient group that received stem cells from matched unrelated donors and was directly linked with a faster T cell recovery and higher T cell chimerism from reconstituted HSCT grafts. About 4/9 patients were alive in this study with a median follow-up of 23.1 months (56). Lee et al. reported results from a Phase I study in patients with AML, myelodysplastic syndromes (MDS), and chronic myelogenous leukemia (CML), in which alloreactive haploidentical NK cells (CD3 depleted and CD56 selected) were administered along with IL-2 injections, followed by thymoglobulin conditioning and allogeneic HSCT. Thymoglobulin was administered to prevent NK cells from hampering engraftment of allogeneic HSCT. Out of 20 evaluable patients, 16 had GvHD (10/16 aGvHD and 6/16 cGvHD) after transplantation. In this study, GvHD was not directly associated with donor T cell or NK cell contents. From this study, also it was concluded that the lack of anti-leukemic effect was mainly due to the low dose of infused NK cells and it was further suggested that thymoglobulin conditioning could also have potentially affected NK cells survival *in vivo* (57).

Later, Choi et al. presented results from a modified treatment protocol of four consecutive infusions of *ex vivo* activated and expanded haploidentical NK cells after HLA-matched HSCT and compared the outcomes to their previous study, in which they administered two infusions. In the subsequent study, additional donor NK-cell infusions were given on days 6 and 9 (i.e., at days 6, 9, 13, and 20). Out of 51 patients with ALL (*n* = 6) and AML (*n* = 45), 24/51 (47%) had four NK infusions. Out of 45 evaluable patients, the 3-year OS rate was 9% in AML and 21% for ALL and 9/45 had aGvHD. Early administration of NK cells after HSCT caused significant toxicities with no improvements in anti-leukemic effects, compared to the previous study. In this study group, a higher CR rate correlated with higher expression levels of NK activating receptors NKG2D and NCRs (NKp44, NKp46, and NKp30) on donor NK cells. In addition, NKp30 expression was significantly higher than that of NKG2D and other NCRs, thus suggesting a role for NKp30 as a predictive biomarker for anti-leukemic effects of NK cells (58).

In 2017, Shah et al. published data from a Phase I study, treating MM patients with UCB-derived NK cells (day 5) along with autologous-HSCT (day 0), following high-dose chemotherapy and low-dose lenalidomide. Mononuclear cells (MNCs) isolated from UCB units (CD3 depleted) were cultured with K562-based artificial antigen presenting cells (aAPCs) expressing membrane bound IL-21. No treatment related toxicities or GVHD was reported in this study. During a median follow-up of 21 months in 12 patients, 4/12 patients had progressive disease (PD) or relapsed. Stable expression of NKG2D and increased expression of CD16 and NKp30 of UCB-NK cells were observed in six patients. This study further reiterates the safety of NK-cell infusions in high doses; however, due to combinatorial set up with HSCT and lenalidomide, it is difficult to interpret the clinical efficacy of UCB-NK cells alone from this study (59).

Taken together, it is evident from these studies, as well as from many others, that GvHD, which is mainly caused by T cells from transplanted grafts, is a major concern in the field of allogeneic HSCT. Under these circumstances, it is difficult to reliably study the safety of allogeneic NK-cell infusions. The timing of NK-cell infusion, NK cell dosage and NK cell promoting conditioning regimens are critical factors that need to be more extensively studied to assess the safety and efficacy of allogeneic NK-cell infusions.

## Adoptive NK Cell Therapy in a Non-Transplant Setting

To gain a better understanding of the safety and efficacy of allogeneic NK cell transfer, investigators started to study NK cells in a non-transplant setting. Landmark clinical trials were performed by Miller et al. (50), Iliopoulou et al. (60), Rubnitz et al. (61), Bachanova et al. (62) Curti et al. (63), and Geller et al. (33) predominantly in hematological malignancies, but also in various solid tumors. These studies demonstrated the safety and in part the efficacy of allogeneic NK-cell infusions in the absence of GvHD. A summary of allogeneic NK cell clinical trials in a non-transplant setting with published results is presented in Table 2.

**Table 2:** **Summary of allogeneic NK cell clinical trials in a non-transplantation setting**.

Study	Malignancy	Clinical Trial design	Culture method^a^	Infused dose NK cells	Final product characteristics	Outcome
Phase I (EudraCT number: 2010-018988-41) Dolstra et al. (73)	AML (*n* = 10)	Conditioning with Cy/Flu followed by KIR-mismatched UCB-NK-cell infusion	*Ex vivo* expanded, differentiated and activated UCB-NK cells from unrelated donors. Culture duration: 42 days with GM-CSF, G-SCF, IL-6, SCF, Flt3L, TPO, IL-7, IL-2, and IL-15 ^a^CD34+ selected HSPC’s	Escalating doses: 3 × 10^6^ cells/kg (cohort 1), 10 × 10^6^ cells/kg (cohort 2), and 30 × 10^6^ cells/kg (cohort 3)	Mean purity: 74 ± 13% CD34+ cell product. 75 ± 12% generated CD56+ CD3− NK cells. 0.03 ± 0.04% CD3+ cells. 0.16 ± 0.21% CD19+ cells. Mean viability: 94%	Well tolerated, no GvHD nor toxicity. 4/10 DFS for 55, 47, 17, and 12 months after infusion
Phase I (NCT01898793) Romee et al. (72)	AML (*n* = 13)	Conditioning with Cy/Flu followed by cytokine-induced memory-like NK-cell infusion and subsequent IL-2 therapy (every other day, 6×)	*Ex vivo* expanded and activated PBNK cells from haploidentical donors. Culture duration: 12–16 h with IL-15, IL-12, and IL-18. ^a^CD3 depleted and CD56 selected	Repeated dose: level 1: 0.5 × 10^6^ NK cells/kg level 2: 1 × 10^6^ NK cells/kg level 3: 10 × 10^6^ NK cells/kg	Purity: >90% CD56+ CD3− cells	Well tolerated, no GvHD. 4/13 NE, 4/13 TF-PD, 3/13 CR, 1/13 Cri, and 1/13 MLFS
Phase I (NCT00799799) Curti et al. (71)	AML (*n* = 16)	Conditioning with Cy/Flu followed by KIR ligand-mismatched NK-cell infusion; IL-2 therapy (3× weekly for 2 weeks)	PBNK cells from haploidentical donors. ^a^CD3 depleted and CD56 selected	Single dose: 1.29–5.53 × 10^6^ cells/kg	Median purity: infused CD3+ cells: 0.65 × 10^5^ cells/kg. Mean viability: 95%	Feasible study, moderate toxicity. 9/16 DFS, 7/16 in relapse (3–51 months), 1/16 died of bacterial pneumonia
Phase II (NCT00526292) Shaffer et al. (70)	AML (*n* = 6) and MDS (*n* = 2)	Conditioning with Cy/Flu followed by HLA-mismatched NK-cell infusion; IL-2 therapy (6×) starting 1 day before and after NK-cell infusion	PBNK cells from haploidentical donors. ^a^CD3 depleted and CD56 selected	Single dose: 4.3–22.4 × 10^6^ cells/kg	Purity: ≥90% CD3− CD56+ cells. CD3+ cells <0.1%. Viability: 82–100%	No GvHD. 3/8 PR, 5/8 no response. Median survival is 12.9 months
Phase I (NCT01212341) Yang et al. (69)	Lymphoma (*n* = 2) and solid tumor (*n* = 18)	KIR ligand-mismatched NK-cell infusion	*Ex vivo* expanded and activated PBNK cells from unrelated donors. Culture duration: 14 days with irradiated auto-PBMCs, OKT3 and IL-2	Single dose: 1 × 10^6^ cells/kg (cohort 1) 1 × 10^7^ cells/kg (cohort 2) Repeated dose: 1 × 10^6^ cells/kg (cohort 3) 3 × 10^6^ cells/kg (cohort 4) 1 × 10^7^ cells/kg (cohort 5), and 3 × 10^7^ cells/kg (cohort 6)	Purity: CD16 +/CD56+ cells: 98.13 ± 1.98%; CD3+ cells: 0.41 ± 0.43%; CD14+ cells: 0.40 ± 0.37%; CD19+ cells: 0.15 ± 0.25%. Fold expansion: 757.5 ± 232.2. Viability: 92.9 ± 2.1%	No GvHD nor severe toxicities. 8/20 SD, 9/20 PD, 3/20 NE. Median PFS in SD patients: 4 months (2–18 months)
Phase I (NKAML: NCT00697671) Pilot study (NKHEM: NCT00187096) Rubnitz et al. (67)	Relapsed leukemia post HSCT (*n* = 15) Refractory/relapsed leukemia (no prior HSCT) (*n* = 14)	Conditioning with Clo/Eto/Cy followed by KIR-matched or -mismatched NK-cell infusion; IL-2 therapy (6×) starting 1 day before and after NK-cell infusion	*Ex vivo* expanded PBNK cells from haploidentical donors. Culture duration: >12 h. ^a^CD3 depleted and CD56 selected	Single dose: 3.5–103 × 10^6^ cells/kg	Median purity: 98.4% CD56+ cells. 0% CD3+ CD56− T cells. 0.31% CD19+ B-cells	Well tolerated, no GvHD. 6/29 PR, 14/29 CR, 8/29 no response, and 1/29 NE. 4/29 are alive and DFS
Phase I (EudracT number: 2005-006087-62) Kottaridis et al. (66)	AML (*n* = 7)	Conditioning with Flu and TBI followed by haploidentical tumor primed NK-cell infusion	*Ex vivo* expanded and activated PBNK cells from haploidentical donors. Culture duration: o/n with CTV-1 lysate and cryopreserved for infusion. ^a^Only CD56 selected	Single dose: 1 × 10^6^ cells/kg	Purity: CD56+ cells 97.17% of which 80% CD56+ CD3− cells	Serious adverse reactions, no GvHD. 3/7 in CR remained in remission, 1/7 in PR achieved CR, 2/7 relapsed and 1/7 died (6 months follow-up). Median OS: 141–910 days
Phase I (BB-IND-14560) Szmania et al. (65)	MM (*n* = 8)	Conditioning with Bor (+/−Cy/Flu/Dex) followed by fresh haplo-(*n* = 6) or cryopreserved auto (*n* = 2) NK cells	*Ex vivo* expanded and activated PBNK cells from haploidentical (fresh) and autologous (cryopreserved) donors. Culture: 8–9 days with K562-mb15-41BBL stimulator cells and IL-2	Single dose: 2 × 10^7^–1 × 10^8^ cells/kg	Median purity: 78% CD3− CD56+ cells. CD3+/CD56– 0.1%. Viability cryopreserved: 94%. Viability fresh: 93%. Recovery cryopreserved: 16%. Recovery fresh: 119%	Feasible and safe. 1/8 PR, 6/8 PD, 1/8 NE, and 3/8 died between days 11 and 98 after NK-cell infusion
Phase II (NCT00274846) Bachanova et al. (64)	AML (*n* = 57)	Conditioning with Cy/Flu; IL2DT in cohort 3 followed by haploidentical NK-cell infusion 1 day later; IL-2 therapy (14×, daily)	*Ex vivo* expanded and activated PBNK cells from haploidentical donors. Culture duration: o/n with IL-2. ^a^CD3 depleted (cohort 1) or CD3 depleted/CD56 selected (cohort 2) or CD3/CD19 depleted (cohort 3)	Single dose: 0.96 ± 0.3 × 10^7^ cells/kg (cohort 1) 0.34 ± 0.05 × 10^7^ cells/kg (cohort 2) 2.6 ± 1.5 × 10^7^ cells/kg (cohort 3)	Purity: NK cells 39 ± 9%, T cells: 0.7% (cohort 1) NK cells 75 ± 6%, T cells: 1.3% (cohort 2) NK cells 54 ± 16%, T cells: 0.3% (cohort 3)	Well tolerated, no GvHD and mild toxicities. 9/42 in remission (1.8–15 months) (cohorts 1 and 2, *n* = 42). 8/15 in remission (1–32 months) (cohort 3, *n* = 15). DFS: 5% (cohorts 1 and 2) and 33% in cohort 3
Tonn et al. (43)	Solid tumors/sarcoma (*n* = 12) Leukemia/lymphoma (*n* = 2)	Pretreatment with mPred following NK-92 cell infusion	*Ex vivo* expanded and activated allogeneic NK-92 cells. Culture duration: 100–300 h with IL-2. ^a^No selection	Repeated doses (2 × 48 h apart): 1 × 10^9^ (cohort 1), 3 × 10^9^ (cohort 2) and 1 × 10^6^ (cohort 3) cells/m^2^ and additional dose level of 10^10^ cells/m^2^ in some patients	Viability: >80%. Fold expansion: 32	Infusion of 10^10^ NK-92 cells/m^2^ were well tolerated. 12/15 PD, 2/15 MR, 1/15 SD for 2 years, OS: 13–801 days
Pilot study (NCT00799799) Curti et al. (63)	AML (*n* = 13)	Conditioning with Cy/Flu followed by KIR ligand-mismatched NK-cell infusion; IL-2 therapy (3× weekly for 2 weeks)	PBNK cells from haploidentical donors. ^a^CD3 depleted and CD56 selected	Single dose: 1.11–5 × 10^6^ CD3− CD56+ cells/kg	Mean viability: 95%. Median purity: 93.5% NK cells. Maximum T-cell dose 10^5^ cells/kg	Feasible and safe, no GvHD. 5/13 active disease: 1/5 CR (6 months), 4/5 died of PD. 3/6 treated in CR are DFS (34, 32, and 18 months), 2/13 in MR in CR (4 and 9 months)
Phase II (BB-IND 8847) Geller et al. (33)	Refractory metastatic breast cancer (*n* = 14) Ovarian cancer (*n* = 6)	Conditioning with Cy/Flu with or without TBI followed by allogeneic NK-cell infusion; IL-2 therapy (3× weekly for 2 weeks)	*Ex vivo* expanded and activated PBNK cells from haploidentical donors. Culture duration: o/n with IL-2	Single dose: 8.33 × 10^6^–3.94 × 10^7^ cells/kg	Viability: >70%. Median T cells: 0.11% CD3+ cells	TLS and PLS and limited infusion or IL-2 related toxicities. 1/20 died due to grade 5 toxicity. 4/20 PR, 12/20 SD, and 3/20 PD (between 31 and 109 days)
Pilot study Bachanova et al. (62)	B-cell NHL (*n* = 6)	Conditioning with Cy/Flu and mAb (rituximab, 4×) before and after haplo NK-cell infusion followed by IL-2 therapy (6×, every other day)	*Ex vivo* expanded and activated PBNK cells from haploidentical donors. Culture duration: 8–16 h with IL-2	Single dose: 21 ± 19 × 10^6^ NK cells/kg	Purity: 43 ± 11% NK cells. 0.16 ± 0.12% T cells	Feasible and safe. 2/6 CR, 2/6 relapsed at 6 months, 2/6 died
Pilot study NKAML Rubnitz et al. (61)	AML (*n* = 10)	Conditioning with Cy/Flu followed by KIR-mismatched NK-cell infusion; IL-2 therapy (6×) starting 1 day before and after NK-cell infusion	PBNK cells from haploidentical donors. ^a^CD3 depleted and CD56 selected	Single dose: 5–81 × 10^6^ cells/kg	Median purity: B-cells 0.097 × 10^6^ cells/kg. T cells 1 × 10^3^ cells/kg	Feasible and safe. 10/10 in remission (569–1,162 days)
Phase I (EudraCT number: 2005-005125-58) Iliopoulou et al. (60)	Non-SCLC (*n* = 16)	Haploidentical NK-cell infusion after chemotherapy	*Ex vivo* expanded and activated PBNK cells from haploidentical donors. Culture duration: 21–23 days with IL-15 followed by 1 h with IL-15 and hydrocortisone. ^a^Only CD56 selected	Repeated doses (2–4): 0.2–29 × 10^6^ cells/kg per dose	Median purity: (T cells) CD3+ CD56+ CD28− 0.12 × 10^6^ cells/kg. CD56+ CD3 cells 97.9% (after 20 days culture). Fold expansion: 23	Safe, no GvHD. 2/16 PR, 6/16 SD, 7/16 PD, 1/16 not treated. 1-year OS 56% (9/16), 2-year OS 19% (4/16)
Phase I Arai et al. (42)	Metastatic RCC (*n* = 11) or Malignant Melanoma (*n* = 1)	NK-92-cell infusion	*Ex vivo* expanded and activated allogeneic NK 92 cells. Culture duration: 15–17 days with or without IL-2. ^a^No selection	Repeated doses (3× in cohort): 1 × 10^8^ (cohort 1), 3 × 10^8^ (cohort 2), 1 × 10^9^ (cohort 3), and 3 × 10^9^ (cohort 4) cells/m^2^	Fold expansion: 200 over 15–17 days. Viability: ≥80%	Safe and feasible, mild toxicities (1 grade 4, hypoglycemia). 10/12 PD (died between day 101 and 1,059), 1/12 alive (1,450 days) and 1/12 died of bronchopneumonia (day 832)
Phase I (BB-IND 8847) Miller et al. (50)	Metastatic Melanoma (*n* = 10), Metastatic RCC (*n* = 13), Refractory HL (*n* = 1), and AML (*n* = 19)	Conditioning with low Cy/mPred or Flu or high-Cy/Flu followed by NK-cell infusion; IL-2 therapy (14×, daily)	*Ex vivo* expanded and activated PBNK cells from haploidentical donors. Culture duration: o/n with IL-2	Escalating doses: low cy/mPred: 1 × 10^5^, 1 × 10^6^, 1 × 10^7^, or 2 × 10^7^ cells/kg (at least three per cohort). Flu or high-Cy/Flu: 2 × 10^7^ cells/kg	Viability: >70%. Purity: NK cells 40 ± 2%. T cells 1.75 ± 0.3 × 10^5^ cells/kg is 0.9 ± 0.1%. Monocytes 25 ± 1.6% and B-cells 19 ± 2%	Feasible and tolerated without toxicities. Low-Cy/mPred: 2/17 with MRCC SD for 20 and 21 months. 4/17 with MM SD for 4–9 months (*n* = 17) High-Cy/Flu: 5/19 AML pts in CR (*n* = 19)

*CNS, central nervous system; MPDs, myeloproliferative disorders; LPD, lymphoproliferative disorder; MM, multiple myeloma; MDS, myelodysplastic syndromes; MDN, myelodysplastic neoplasms; MPN, myeloproliferative neoplasms; AML, acute myeloid leukemia; LBLL, lymphoblastic leukemia-lymphoma; ALL, acute lymphoblastic leukemia; NB, neuroblastoma; RMS, rhabdomyosarcoma; CML, chronic myelogenous leukemia; NHL, non-Hodgkin’s lymphoma; MCL, mantle cell lymphoma; DLBCL, diffuse large B cell lymphoma; FL, follicular lymphoma; ALCL, anaplastic large cell lymphoma; HL, Hodgkin’s lymphoma; RCC, renal cell cancer; SCLC, small cell lung cancer; CLL, chronic lymphocytic leukemia; HCC, hepatocellular carcinoma; PNET, primitive neuroectodermal tumor; ACC, adrenal cortical carcinoma; EWS, Ewing sarcoma; DSRCT, desmoplastic small round cell tumor; Cy, cyclophosphamide; Flu, fludarabine; Bor, bortezomib; Dex, dexamethasone; Clo, clofarabine; Eto, etoposide; Cis, cisplatin; Pac, paclitaxel; Doc, docetaxel; Vin, vinorelbine; Gem, gemcitabine; Car, carboplatin; Pem, pemetrexed; TBI, total body irradiation; Tac, tacrolimus; Pred, prednisolone; mPred, methylprednisolone; Thy, thymoglobulin; Vin, vincristine; Adr, adriamycin; Predn, prednisone; Mel, melphalan; OKT3, muromonab-CD3; HSPC, human stem and progenitor cells; aGvHD, acute graft versus host disease; DNKI, donor NK-cell infusion; Stim, stimulated; Unstim, unstimulated; DFS, disease-free survival, CR, complete remission; PR, partial response; MR, minimal response; SD, stable disease; PD, progressive disease; aNK-DLI, donor-derived IL-15/4-1BBL activated NK-cell infusion; TRM, transplant-related mortality; MC, mononuclear cell; TLS, tumor lysis syndrome; PLS, passenger lymphocyte syndrome; NE, not evaluable*.

*^a^Culture method displays CD3 depleted PBMC’s, otherwise deviated selection method is mentioned in product characteristics*.

Here, we focus on the latest reports from clinical trials using allogeneic NK cells in a non-transplant setting. Bachanova et al. developed a recombinant cytotoxic protein, i.e., an IL-2/diphteria toxin fusion protein (IL2DT), which functions by selectively depleting the IL-2 receptor CD25 expressing cells, including regulatory T cells (T-regs). In total, 57 AML patients were treated with KIR and HLA-mismatched haploidentical NK cells and 15 of them in cohort 3 received IL2DT, 1 or 2 days prior to NK-cell infusion, to deplete T-regs. In addition to IL2DT treatment, three different processing methods were used, i.e., a CD3-depleted cohort (cohort 1, *n* = 32), a cohort using CD3 depletion followed by CD56 selection (cohort 2, *n* = 10), and a cohort using CD3 and CD19 depletion (cohort 3, *n* = 15). Higher NK cell doses were obtained from cohort 3, and it could possibly be the reason for the observed longer disease-free survival (DFS) (33% versus 5% at 6 months) in cohort 3 compared to cohorts 1 and 2. In this study, endogenous IL-15 serum levels correlated with reduced T-reg levels in patients treated with IL2DT (64).

Szmania et al. investigated the effect of infusing cryopreserved and freshly prepared NK cells (CD3 depleted), either allogeneic or autologous, given after bortezomib with or without lymphodepletion in high risk relapsed MM patients. NK cells were cocultured with K562-mb15-41BBL cells for 8–9 days. Initially, 6 out of 8 NK products were cultured with low dose IL-2 (10 U/ml), however, increasing the dosage of IL-2 to 500 U/ml resulted in enhanced expression of NKp30 and NK cytolytic activity without affecting the T cell content of the NK cell product and, therefore the last 2 patients in this study were treated with high dose IL-2 cultured NK cells. The highest post-transfer number of circulating NK cells was observed in the high dose IL-2 group. Patients treated with fresh NK cells showed a median 21-fold increase in peripheral NK cell rates by day 7, while no *in vivo* expansion of NK cells was seen in patients treated with cryopreserved NK cells. Overall, the NK-cell infusions were well tolerated and no GvHD was observed. From 7 evaluable patients, 6 had (PD), and 1 had a partial response (PR) for up to 6 months post infusion (65).

In the same year, Kottaridis et al. presented data from a clinical trial in AML, for the first time using tumor-primed NK cells from related haploidentical donors. During a 6-month follow-up period (*n* = 7), three patients in CR remained in CR, one patient in PR achieved CR, two patients relapsed, and one patient died. Median OS was 468 days post NK-cell infusion (66).

Rubnitz and his team reported on the safety and feasibility of haploidentical NK cell therapy in children with relapsed or refractory leukemia. NK cells were administered along with IL-2 injections in 29 patients, out of which 14 had not undergone HSCT (cohort I) and the other 15 have relapsed after HSCT (cohort II). In total, 90% of the NK cell donors were KIR mismatched and when the outcomes from both cohorts were combined, 14/29 were in CR, 6/29 showed PR, and 8/29 patients showed no response to the treatment (67).

The first clinical trial to adoptively transfer allogeneic NK cells derived from peripheral blood of unrelated donors without immune suppression was performed by Yang et al. In this study, allogeneic IL-2-activated NK cells (MG4101) were expanded at Green Cross Lab Cell (68) and administered to patients with advanced lymphoma and recurrent solid tumors. Following NK cell-adoptive transfer, an increase in NKG2D expression levels on CD8^+^ T cells, a reduction in the number of T-regs, and MDSCs followed by a decrease in serum levels of transforming growth factor-beta were noted. An enhanced PFS was noted in KIR-mismatched NK cell recipients. In addition, a KIR B haplotype was associated with a higher incidence of stable disease (SD). This study demonstrated that KIR-ligand mismatched donor NK cells can be safely administered without any sign of GvHD and with a GvT effect. Though an antitumor effect of the adoptively transferred NK cells could be observed, their persistence *in vivo* was shorter (between 1 and 4 days) in comparison to other clinical trials. This stresses the potential need for an effective NK cell-promoting conditioning regimen, to increase the life span and migration of NK cells in patients (69).

Shaffer et al. published results from a Phase II study in patients with relapsed or progressive AML (*n* = 6) or MDS (*n* = 2), treated with allogeneic NK-cell infusions and supported by IL-2 injections *in vivo*. NK cell donor chimerism was not detected post infusion, and no signs of GvHD were reported from this study. About 3/8 patients achieved PR of which 1/6 patients with AML and 1/2 patients with MDS achieved a CR after treatment but relapsed within 2 months. Of note, both these patients survived for 20.2 months post NK infusion, while the remaining 5 patients without response had a median survival of 5.4 months (70).

Around the same time, Curti et al. published results from a Phase I trial using KIR ligand-mismatched haploidentical NK cells to treat AML patients in CR (*n* = 16). About 7/16 patients relapsed, while 9/16 remained disease free at a median follow-up of 22.5 months. Overall, 69% (11/16) responded to therapy, with no signs of GvHD. Prolonged DFS was higher in patients with an absolute increase in the number of circulating alloreactive NK cells in this study (71).

Romee et al. investigated the antitumor effects of cytokine induced memory-like NK cells, which were adoptively transferred in relapsed or refractory AML patients after overnight activation with IL-12, IL-15, and IL-18. They were defined as memory-like NK cells based on their enhanced responsiveness upon restimulation with cytokines. NK-cell infusions were safe and well tolerated and no GvHD was reported in this study. Out of nine evaluable patients, four had CR and five showed disease responses (72).

Most recently, a first clinical trial using UCB CD34+ progenitor cell-derived NK cells was published by Dolstra et al. Allogeneic NK cells were generated from UCB CD34+ cells using an *ex vivo* expansion and differentiation method developed by Glycostem Therapeutics (41). In this study, 10 AML patients in morphologic CR, who were ineligible for HSCT transplantation, received partially HLA-matched (5/10 KIR ligand-ligand mismatched and 7/10 KIR receptor-ligand mismatched) UCB-NK cells (oNKord^®^). Following Cy/Flu conditioning, lymphocytopenia was induced and found to correlate with elevated IL-15 levels, which peaked at day 6 after NK-cell infusion. Out of 10 treated patients, 5 were alive and 4 had a DFS of 60, 52, 22, and 16 months after infusion. About 2/4 patients with very poor prognosis (i.e., with detectable MRD in bone marrow before NK infusion) became MRD negative for 6 months post NK infusion, indicating a potential GvT effect. UCB-NK-cell infusions were safe and well tolerated without signs of GvHD. Interestingly, UCB-NK cells expressing low levels of KIRs and CD16a at the end of the *ex vivo* culture, underwent further maturation post-transfer *in vivo*, resulting in the upregulation of KIRs and CD16a, but continued to preserve the activated phenotype denoted by high expression of NKp30, NKp44, NKp46, NKG2D, and DNAM (73).

In addition to NK cells from PBNK and UCB-NK, two clinical studies reported on the use of the NK-92 cell line. A Phase I trial conducted by Arai et al. investigated the safety and feasibility of allogeneic NK-92 cells in advanced renal cancer and melanoma. A total of 12 patients were evaluated and 6/12 had PD, 4/12 had SD, while 1/12 had minor response and 1/12 had mixed response, 4 weeks post infusion (42). Similarly, in another study with NK-92 conducted by Tonn et al., 15 patients were included with advanced solid (*n* = 13) and hematological malignancies (*n* = 2). About 1/7 tested patients produced antibodies against the HLA antigens expressed by NK-92 cells, 1/15 showed SD, 1/15 a mixed response, and the rest of the patient group had disease progression, being treated with a maximum tolerated dose of 10^10^ cells/m^2^. NK-92 cells had a very short persistence (48 h) *in vivo* (43). As NK-92 cells are derived from cancer (lymphoma) cells and require irradiation before infusion, which could hamper their ability to proliferate and home *in vivo*, potentially limiting their efficacy.

Overall, analyzing the data from adoptive allogeneic NK cell therapy trials in a non-transplant setting, we conclude that such treatments are very safe and well tolerated and efficacious in hematological malignancies, especially in AML, but as yet relatively ineffective in solid tumors. Trials using allogeneic NK cells alone yielded valuable information on the *in vivo* persistence, donor chimerism, and antitumor potential in different indications. Furthermore, unlike combined approaches with HSCT, the absence of life-threatening GvHD and major treatment-related toxicities makes this method advantageous and provides an opportunity to further enhance the cytotoxic effects of allogeneic NK cells.

## Approaches to Augment NK Cell Functions: A View on Biotech Industries

**Figure 1:**
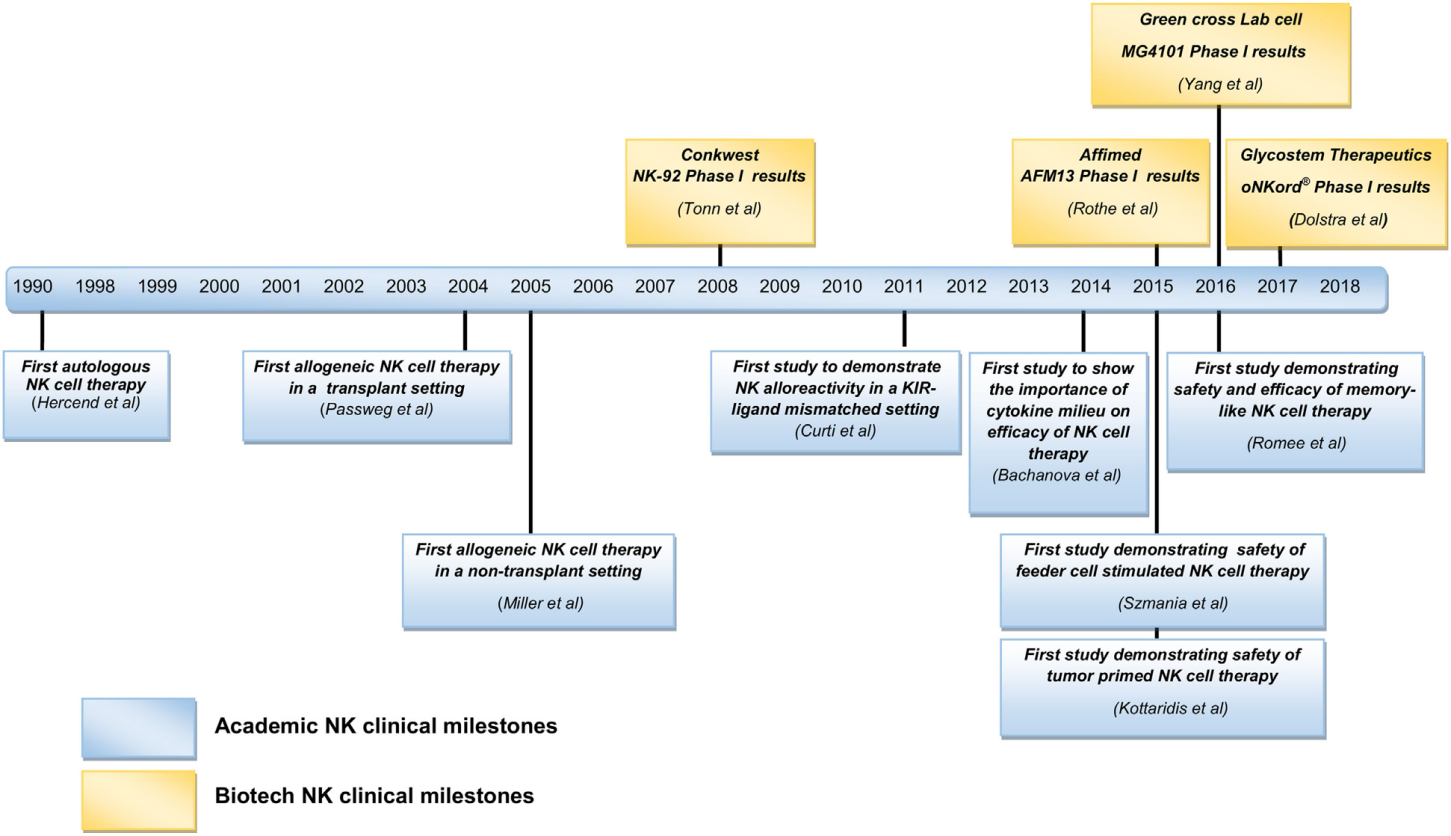
**Summary of natural killer (NK) cell clinical milestones from academia and biotech industries**.

As reviewed above, various clinical trials have been published, mainly initiated by academia, proposing allogeneic NK cells as an effective therapeutic option. As a result of these studies, interest in NK cell-based immunotherapy strategies has been engendered in an increasing number of biotech companies. Clinical trials conducted in academia are often restricted to Phase I or II, as progression of experimental therapies to Phase III clinical trials and further on to commercialization and marketing requires a level of funding that surpasses the capacity of academic institutions. The financing of market enabling studies is coming mainly from industry. Although NK cells can be effective in some types of cancer as a monotherapy, considering their heterogeneity, complex networking, and the inherent adaptability of several tumors to evade killing by immune cells, one believe is that it is necessary to improve on the efficacy of currently available NK cell products. In this respect, it is worthwhile to consider combinatorial approaches of different treatment strategies involving NK cell functions. A summary of biotech companies involved in NK cell research is listed in Table 3 and Figure 1. Here, we review for a selected group of NK cell companies, which develop NK cell-specific treatments, the underlying scientific principles and findings of their product pipelines, revealing highly innovative concepts that herald future clinical applications.

**Table 3:** **List of biotech NK cellular therapies and NK cell function enhancing compounds**.

Company	NK cell product	Product characteristics	Disease target	Product stage
Fortress Biotech Inc.	CNDO-109	Tumor primed NK cells	AML	Phase I/II
Multimmune GmbH	ENKASTIM-ev	A synthetic peptide which mimics Hsp70 and activates NK cells *ex vivo*	Metastatic colon and non-small cell lung cancer	Phase II
Glycostem Therapeutics	oNKord^®^	NK cells derived from umbilical cord blood (UCB) progenitor cells	AML and solid tumors	Phase I (AML)
NantKwest Inc.	Activated NK-92 cells (aNK cell)	IL-2-dependent tumor cell-derived NK cell line	Solid tumors and hematological malignancies	Phase I
	High affinity NK cells (haNK)	aNK cells genetically modified to express CD16 for ADCC with therapeutic mAbs	Ideally in combination with IgG_1_ therapeutic mAbs in solid tumors (e.g., cetuximab) and hematological malignancies (e.g., rituximab)	Preclinical
	Target-activated NK cells (taNK)	aNK cells genetically modified to express CARs	NK-92 CARs are developed targeting tumor antigens in neuroblastoma, melanoma, breast cancer, MM and leukemias	Preclinical
Green Cross Lab Cell	MG4101	*Ex vivo* expanded NK cells derived from CD3 depleted unrelated donors	Solid tumors and lymphoma (NCT01212341)	Phase I
Gamida Cell	NAM-NK cells	Nicotinamide-based PBNK cell culture system	Solid tumors and hematological malignancies	Preclinical
Celgene Cellular Therapeutics	NK cells	NK cells derived from UCB and placenta.	Solid tumors and hematological malignancies	Preclinical
Fate Therapeutics Inc.	iNK cells	NK cells derived from induced pluripotent stem cells	Solid tumors and hematological malignancies	Preclinical
Sorrento Therapeutics Inc	CARs to enhance tumor homing of NK-92 cells	NK-92 cells CAR targeting programming death ligand-1 and NK-92 CAR targeting receptor tyrosine kinase like orphan receptor to increase NK-92 tumor homing	Solid tumors and hematological malignancies	Preclinical
Nkarta Therapeutics	NKG2D CARs	NKG2D CARs developed with NK-92 and PBNK to enhance the functions of NKG2D receptor in NK cells	Osteosarcoma and hepatocellular carcinoma	Preclinical
Ziopharm Oncology Inc.	HLA gene editing	Zinc finger nuclease technology to delete HLA-A sequences from allogeneic NK cells, allowing them to evade recipient T cell killing	Solid tumors and hematological malignancies	Preclinical
**Company**	**NK cell enhancing products**	**Product characteristics**	**Disease target**	**Product stage**
Kyowa Hakko Kirin	Mogamulizumab	Fc optimized anti CCR4 CD20 mAb	CTCL	Phase III
Genentech	Obinutuzumab	Fc optimized anti CD20 mAb	CLL	Phase II
Mentrik Biotech, LLC	Ocaratuzumab	Fc optimized anti CD20 mAb	CLL	Phase II
Roche Glycart	Imgatuzumab	Fc optimized anti EGFR mAb	Head and neck and KRAS mutant colorectal cancer	Phase I/II
Affimed N.V.	AFM13	Bispecific antibody binding to CD16a on NK cells and CD30 on tumor cells	Hodgkin’s lymphoma and lymphomas	Phase II
	AFM22	Bispecific antibody binding to CD16a on NK cells and EGFR vIII on tumor cells	Head and neck and solid tumors	Preclinical
	AFM24	Bispecific antibody binding to CD16a on NK cells and wild type EGFR on tumor cells	EGFR-expressing solid tumors	Preclinical
Innate Pharma S. A.	Lirilumab	mAb to block NK cell inhibitory signaling from KIRs (KIR2DL1–3)	As monotherapy (Phase II, NCT02399917), with nivolumab (Phase I, NCT01592370), with ipililumab (Phase I, NCT01750580), 5-azacytidine (Phase I, NCT02399917), with nivolumab + 5-azacytidine (Phase II, NCT02599649), with elotuzumab (NCT02252263) and with rituximab (Phase I, NCT02481297)	Phase I/II
Innate Pharma S. A.	Monalizumab	mAb to block NK cell inhibitory receptor NKG2A	As monotherapy (Phase I/II, NCT02459301, NCT02331875) with cetuximab (NCT02643550), with ibrutinib (NCT02557516) and with durvalumab (NCT02671435)	Phase I/II
	IPH4102	mAb to block NK cell inhibitory receptor KIR3DL2	As monotherapy in CTCL (NCT02593045)	Phase I
	IPH4301	mAb to target NKG2D ligands MICA/MICB and it also mediates ADCC with NK cells	Solid tumors and hematological malignancies	Preclinical
Altor Biosciences corporation	ALT-803	IL-15 super agonist reported to stably express IL-15. Increases NK cell proliferation *in vivo*, also enhances expansion of migratory NK subsets	Advanced solid tumors (NCT01946789), MM (NCT02099539), HIV patients (NCT02191098), with nivolumab in NSCLC (NCT02523469), with rituximab (NCT02384954) in B cell Non-Hodgkin Lymphoma (NHL) (NCT02384954), with (BCG) in Non-Muscle Invasive Bladder Cancer (NCT02138734), with chemotherapy drugs gemcitabine and Nab-paclitaxel in advanced pancreatic cancer (NCT02559674)	Phase I/II
NOXXON Pharma	NOX-A12	Functions as chemokine receptor CXCL12 inhibitor, enables the release of CXCL12 from the surface of tumor stromal cells, thus facilitating migration of tumor cells toward NK cells	Solid tumors and MM	Preclinical
AvidBiotics	MicAbody proteins	Dual role: binds to NKG2D receptor in NK cells and to target antigens of interest simultaneously	Solid tumors and hematological malignancies	Preclinical

*KIRs, killer cell immunoglobulin-like receptors; ADCC, antibody-dependent cell mediated cytotoxicity; aNK, activated NK cells; haNK, high affinity NK cells; taNK, target activated NK cells; mAbs, monoclonal antibodies; NAM, nicotinamide; CARs, chimeric antigen receptors; EpCAM, epithelial cell adhesion molecule; AML, acute myeloid leukemia; KIR2DL1–3, killer cell immunoglobulin like receptor two domains long cytoplasmic tail 1–3; KIR3DL2, killer cell immunoglobulin like receptor three domains long cytoplasmic tail 2; MICA/MICB, MHC class-I-chain related protein A and B; HIV, human immunodeficiency virus; NSCLC, non-small-cell lung cancer; BCG, Bacillus Calmette Guerin; MM, multiple myeloma; CLL, chronic lymphocytic leukemia; CTCL, cutaneous T cell lymphoma; EGFR, epidermal growth factor receptor*.

## Fc Optimized Monoclonal Antibodies (mAbs)

The potential of NK cells to mediate ADCC with therapeutic mAbs has been well described over the years (74). However, concerns have been voiced based on results from certain clinical trials, showing that polymorphisms in NK CD16 (V158V, V158F, and F158F) could influence the efficacy of mAb treatment and ADCC (75). To address this issue and limit the variations between different CD16 sequences, Fc glyco-engineered (defucosylated) mAbs with enhanced binding affinities to NK CD16a were developed. The Fc optimized anti-CCR4 mAb mogamulizumab (76) (Kyowa Hakko Kirin) has entered Phase III clinical testing in patients with adult T cell leukemia, emerging as the lead NK cell ADCC product to reach the market soon. Fc-optimized anti-CD20 mAbs Obinutuzumab (Genentech) (77) and Ocaratuzumab (Mentrik Biotech, LLC) (78) are currently tested in patients with chronic lymphocytic leukemia and follicular lymphoma. Similarly, the Fc-optimized anti-EGFR mAb imgatuzumab (Roche Glycart) is tested in Phase I/II clinical trials for head and neck cancer and in KRAS mutant colorectal cancer (79, 80). Although Fc-engineered mAbs address NK-mAb-binding issues, reports of serious side effects, like from the imgatuzumab study (81), have made the scientists rethink this strategy and call for the careful study of the advantages and disadvantages of this approach.

## Bispecific Antibodies

In the last decade, several bispecific and trispecific Ab platforms, simultaneously targeting immune cells and tumor cells, have been developed in the field of cancer immunotherapy (82). To date, the majority of bispecific Abs that has been developed targets T cells, while only a limited number of bispecific approaches targets NK cells (83). Affimed is a clinical stage pharmaceutical company developing bifunctional antibodies that recruit immune cells such as T and NK cells to tumor sites. These bispecifics (TandAbs) are tetravalent in nature, thus offering four binding sites, two aimed at tumor antigens and two aimed at immune cells. Currently, Affimed’s AFM13 that targets CD30 on cancer cells and CD16a on NK cells is in clinical Phase II testing in patients with HL. In Phase I studies AFM13 was found to be safe and well tolerated and resulted in an overall response rate of 23%. Furthermore, AFM13 treatment resulted in an increase in NK cell activation and a decrease in soluble CD30 levels in peripheral blood (NCT01221571) (84). Further, two other bispecific CD16a-based tumor targeting antibodies are in preclinical phase development, i.e., AFM22 and AFM24 that bind to EGFRvIII expressed by several solid tumors, including glioblastoma (GBM), and wild-type EGFR, respectively. Another promising NK cell-focused bispecific platform is developed by AvidBiotics to target tumors that evade NK killing *via* downregulation or shedding of the NKG2D ligand MICA, which is a major limiting step in NK-mediated tumor targeting. To overcome this, AvidBiotics designed MicAbody proteins that bind to the NK cell NKG2D receptor with high affinity. Further, this MicAbody was engineered with an additional binding site to target tumor antigens of interest, thus enabling recruitment of NK cells to tumors (85).

## NK Cell Checkpoint Inhibitors

Another strategy to increase NK cell functionality is the disruption or blocking of NK inhibitory signals. Innate Pharma is a clinical stage pharmaceutical company focused on developing NK cell checkpoint inhibitors. Lirilumab (IPH2102/BMS 986015) is a fully humanized IgG_4_ anti-KIR mAb against the inhibitory KIRs KIR2DL1, L2, and L3, which are expressed predominantly on NK cells and on some T cells. Lirilumab induced significant anti-tumor activity of NK cells against HLA-C-expressing tumor cells, contributing to increased survival in lirilumab-treated mice (86). Similar to KIRs, the NK cell inhibitory receptor NKG2A binds to its ligand HLA-E on tumor cells resulting in an inhibition of NK cell function. HLA-E is overexpressed in colon, cervical, and ovarian cancers, thus serving as an escape mechanism for NK killing in these tumors (87, 88). The anti-NKG2A mAb monalizumab was developed to block the interaction between NKG2A and HLA-E and is currently under clinical investigation. IPH4102, which targets KIR3DL2, is under Phase I clinical investigation in cutaneous T cell lymphoma (CTCL). Clinical trials testing lirilumab, monalizumab, and IPH4102 are listed in Table 3.

## Genetic Modification of NK Cells

In addition to successful expansion, differentiation, and demonstrable anti-tumor effects of NK cells, NK cell tumor targeting can be made more specific by employing chimeric antigen receptors (CARs) as demonstrated for T cell adoptive transfer strategies (89). CARs are recombinant Ab-based molecules that upon expression in immune effector cells bind antigens of interest on target cells, resulting in immune activation and enhanced immune effector cell survival through specific intracellular signaling motifs fused to the antigen binding domain [usual a single-chain Fv fragment (scFv)]. PBNK-CARs against breast cancer (HER-2), NB (CD244), and CD19 + B-cell precursor cell ALL (CD19) (90) have demonstrated efficacy in preclinical studies, while two clinical trials are ongoing using modified haplo-identical PBNK cells with anti-CD19 CARs in B cell malignancies (NCT00995137 and NCT01974479) (89). NantKwest, is actively involved in enhancing the functions of its lead product, parental NK-92 cells (activated NK cells, aNK), through gene modifications employing CARs to make them target specific. NK-92 CARs (taNK) are developed against tumor markers in NB (GD2), melanoma (GPA7) (91), breast cancer (EpCAM, HER-2, EGFR) (92, 93), MM [CS1 (94), CD138 (95)], and leukemias (CD19, CD20) (96) and have shown efficacy in preclinical studies. In an alternative approach, NK-92 cells have also been modified to express CD16a (high affinity NK cells, haNK) to promote ADCC (97). NantKwest has also partnered with Sorrento Therapeutics to develop NK-92 CARs targeting programmed death-ligand1 (PD-L1) (98) and receptor tyrosine kinase-like orphan receptor 1 (ROR-1) (99).

Besides specific targeting of tumor antigens and strategies to promote ADCC, Nkarta therapeutics developed NKG2D CARs (NKG2D-CD3ζ-DAP10) using NK-92 cells and PBNK cells, which exhibited enhanced cytotoxicity against osteosarcoma and hepatocellular carcinoma when compared to activated and expanded PBNK cells (100, 101). mRNA-based genetic engineering has been used to enhance migration of NK cells to tumors.

Apart from gene modification, gene editing is also widely used to overexpress or knock out genes of interest to augment NK cell function. Expression of HLA-A on allogeneic NK cells leads to rejection of allogeneic NK cells by the recipient’s T and NK cells. Cooper and colleagues from Ziopharm Oncology used zing finger nuclease (ZFN) technology to remove HLA-A sequences from allogeneic NK cells, thus enabling these immune effector cells to escape rejection from recipient T cells. However, in that case, there is yet a high probability of being attacked by endogenous NK cells targeting HLA-A negative allogeneic cells. This was further addressed by retaining HLA-B and HLA-C genes in donor NK cells (102–104). To increase NK cell persistence *in vivo*, scientists at oNKo-innate identified a group of proteins called suppressor of cytokine signaling (CIS, SOCS 1–7), which negatively regulate CIS pathways. SOCS1 and SOCS3 bind to JAK1, JAK2, and TYK2 molecules and inhibit JAK activity. Similarly, CIS protein binds to JAK1 and suppresses IL-15 signaling in NK cells. It became evident from *in vivo* studies in mice with *Cish^−^*/*^−^* knockout NK cells that loss of CIS led to prolonged IL-15 signaling, resulting in an increased proliferation, survival, and functionality of NK cells (105).

## NK Cells from iPSCs

In recent years, NK cells generated from induced pluripotent stem cells (iPSC-NK) and human embryonic stem cells (hESC-NK) have been gaining more interest as an NK cell therapeutic product. Fate Therapeutics developed a platform technology to generate NK cells from iPSC. hESC/iPSC were made into aggregates by centrifugation to form so-called embryoid bodies (spin EBs) (106), giving rise to hematopoietic progenitor cells expressing CD34 and CD45, which were then differentiated into mature NK cells using a specific cytokine cocktail. iPSC/hESC-derived NK cells were shown to express common NK cell markers, such as KIRs, CD16, NKp44, NKp46, NKG2D, and TRAIL, and were cytotoxic against several hematological and solid tumor cells *in vitro* (107, 108). In the next stage, iPSC/hESC-derived NK cells were successfully expanded using IL-2 and K562-based aAPCs with membrane-bound IL-21 to generate sufficiently high numbers for clinical applications (109).

## NK Cells from Human UCB Cells

Stem cell progenitors from cord blood offer a unique platform to be expanded and differentiated into cytotoxic NK cells. The low immunogenicity of cord blood cells strongly reduces the risk of relapse and GvHD after transplantation (110). Considering the advantages of using cord blood, Glycostem Therapeutics, a clinical stage biotech company, which in the last decade has developed a flexible platform technology to expand and differentiate NK cells from CD34+ cells (111), upgraded this into a large scale GMP UCB-NK platform for clinical implementation (oNKord^®^) (41). UCB-NK cells were infused at up to 30 × 10^6^ cells/kg/bodyweight in elderly AML patients, resulting in excellent safety and initial efficacy in a Phase I trial. Infused oNKord^®^ cells showed active migration to the marrow and further matured in the absence of any exogenous cytokine injections. This confirms previous findings from a preclinical model, showing migration to the bone marrow and upregulation of KIRs and CD16a *in vivo* as well as antileukemic activity (112). oNKord^®^ is well characterized and was found to have a similar functionality and gene expression profile as PBNK cells (113). Furthermore, oNKord^®^ is highly cytotoxic against solid tumor targets such as cervical cancer cells, in which killing was independent of HLA expression levels, tumor histology and HPV types (114), or colorectal cancer cells, in which killing was independent of tumor EGFR levels, and RAS and RAF mutations (115), thus paving the way for oNKord^®^ as immunotherapy for advanced solid tumors.

## Cytokines to Enhance NK Cell Functions

To improve the antitumor activity of autologous NK cells, systemic administration of clinical grade recombinant IL-2 (rIL-2) and single chain IL-15 (scIL-15) has been used in high doses and this has resulted in severe grade 3/4 toxicities (116–118). Since then, their safety and efficacy have been tested in low doses following NK cell-adoptive transfer in cancer patients (50, 63, 119). However, IL-2 resulted in expansion and mobilization of inhibitory T-regs, severely limiting NK cell cytotoxicity (120). This shifted the focus toward the use of IL-15 for clinical trials involving NK cells. Currently more potent and advanced heterodimeric IL-15, which has a longer shelf life than scIL-15, is being tested in several studies (121). IL-15 is known to be more effective in membrane-bound form (i.e., bound to its receptor), engaging target immune cells in a cell contact dependent manner. Campana and his team (from Nkarta Therapeutics) addressed this by stably transducing the membrane bound IL-15 (mbIL-15) gene into proliferating PBNK cells, which were stimulated with K562-mb15-41BBL. mbIL-15 resulted in increased survival, proliferation, and enhanced cytotoxic functions of NK cells (122). Further, Cyto-Sen Therapeutics compared mbIL-15 to K562-based aAPCs with mbIL-21. From their findings, it was evident that mbIL-21 NK cells have a significantly higher expansion and proliferation ability compared to mbIL-15 NK cells (123). Cyto-Sen also developed plasma membrane particles (PM21) engineered from K562-mb21-41BBL cells and found that these PM21 particles stimulated efficient NK cell expansion in AML patient’s PBMC samples (124).

Compared to IL-2, the use of IL-15 minimizes capillary leak syndromes and has less side effects overall, thus providing a strong rationale to use IL-15 instead of IL-2. However, the use of mammalian recombinant IL-15 in the clinic has been limited due to its short half-life and decreased functional activity *in vivo*. Altor BioSciences corporation came up with a unique design to overcome these limitations. It developed an IL-15 super agonist known as ALT-803. It consists of a human IL-15 mutant N72D variant, which is stably complexed with a soluble human IL-15Rα sushi-Fc dimer protein. Enhanced biological activity of ALT-803 was reported in several preclinical studies showing durable antitumor activity in various solid and hematological malignancies (125–128). Furthermore, ALT-803 facilitated expansion of effector and migratory NK cell subsets and significantly decreased the metastatic activity of tumor cells in a murine colon cancer pulmonary metastasis model (129). ALT-803 stimulated primary human NK cells to exhibit increased degranulation, IFNγ production, and ADCC when exposed to B cell lymphoma cell lines coated with IgG_1_ therapeutic anti-CD20 mAbs (130). Several clinical trials are currently ongoing with ALT-803 as monotherapy in patients with advanced solid tumors, hematological malignancies, and AIDS as summarized in Table 3.

## Priming NK Cells to Enhance Tumor Killing

Mark Lowdell and his team proposed that for a NK cell to be able to kill tumor cells, it requires a priming and triggering signal. NK cells failing to kill tumor cells, though they are exposed to the triggering signal, remain inactive due to the absence of a priming ligand. To address this, Fortress Biotech (previously known as Coronado Biosciences) developed a technology to increase NK cell tumor killing using cell lysates from the leukemia cell line CTV-1, known as CNDO-109, to prime NK cells. A Phase I/II clinical trial of activated PBNK cells from haploidentical donors coincubated with CNDO-109, infused at doses of up to 3 × 10^6^ kg/recipient/body weight was tolerable without any adverse reactions. Out of seven evaluable patients, four remained disease relapse free for more than 1 year (131).

Another NK cell-activating product is ENKASTIM-ev, developed by Multimmune GmbH, which mimics the functions of heat shock protein 70 (Hsp70). ENKASTIM-ev resulted in NK specific activation and actively targeted Hsp70 expressing tumors. Safety of Hsp70-activated autologous NK cells has been documented in a Phase I study in patients with metastatic colorectal and non-small cell lung cancer (132).

## Enhancing NK Cell Homing Functions

Gamida-cell developed a feeder cell-free NK cell culture and expansion system containing nicotinamide (NAM) to generate NK cells from PBMC apheresis products. Nicotinamide, a derivative of vitamin B3, serves as a potent inhibitor of NAD dependent enzymes. Results from *in vivo* studies in mice showed that PBNK cells expanded with NAM in feeder free cultures exhibited increased homing potential toward lymphoid organs, with a significant increase in the expression of CD62L (L-selectin) compared to cultures without NAM (133).

## Tumor Disruptive Technology Aiding NK Tumor Recognition

NOXXON Pharma target chemokine receptor CXCL12, with the aim of increasing the sensitivity of tumor cells to drugs and immune cells. Their product NOX-A12 functions as a CXCL12 inhibitor and enabled the release of CXCL12 from the surface of tumor stromal cells and blocked its interaction with cell surface receptors CXCR4 and CXCR7. This mechanism facilitated the mobilization of CXCR4-expressing tumor cells from their tissue niches to areas, where they become easily accessible by NK cells or T cells (134, 135). Using tumor spheroids, increased mobilization of T and NK cells toward tumor cells in the tumor microenvironment was demonstrated. NOX-A12 also enhanced NK killing of obinutuzumab-coated Raji cells *in vitro*, mediated by ADCC (136).

## Conclusion

**Figure 2:**
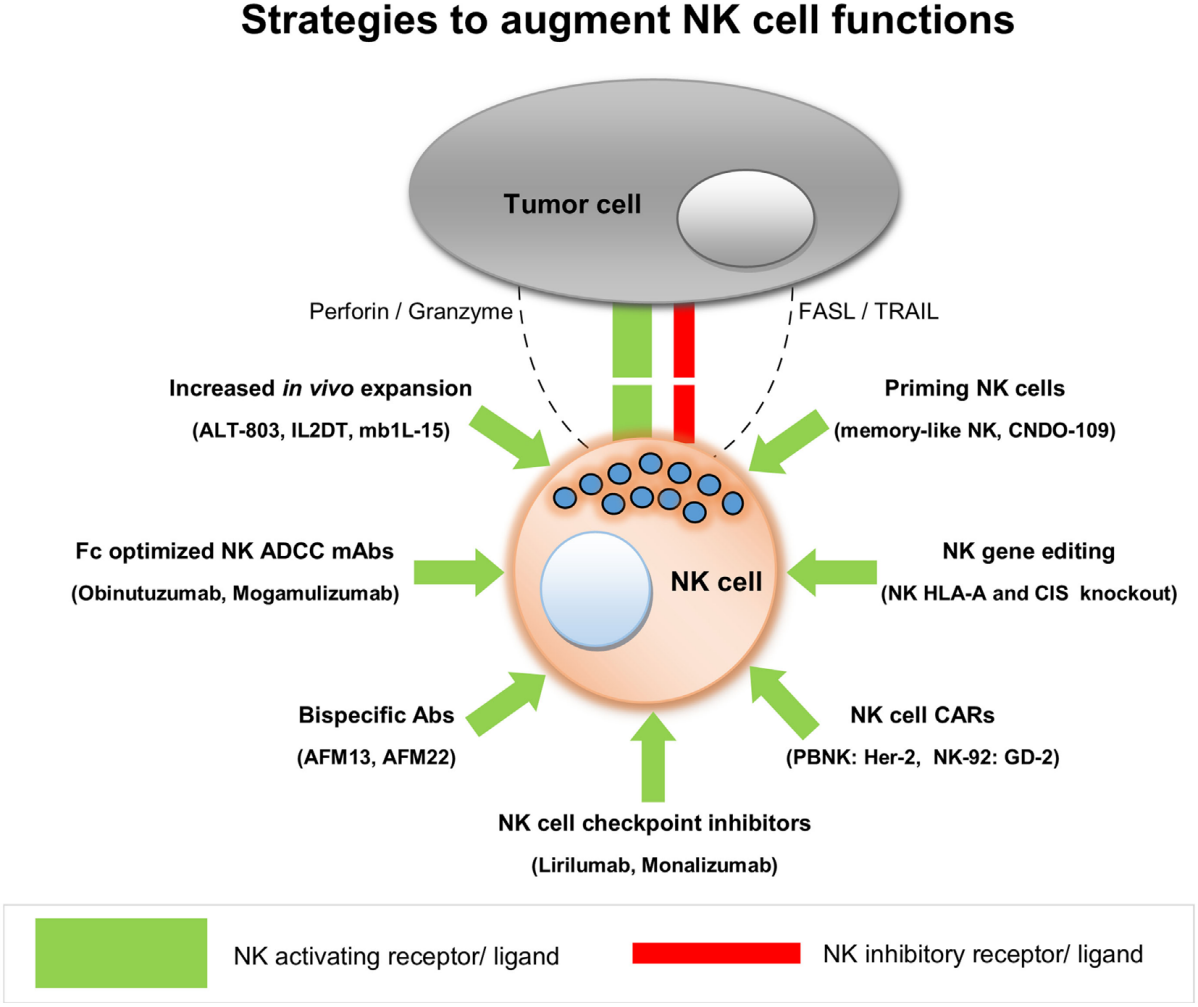
**Strategies to augment natural killer (NK) cell functions**.

From this literature review, we conclude that adoptive transfer of allogeneic NK cells in a non-transplant setting is safe and shows early signs of clinical efficacy against hematological and certain solid tumors. Current data are mostly based on Phase I clinical trials, and hence it is still too early to get an overall picture of NK cell alloreactivity in different kinds of cancer. Most of the clinical studies conducted so far have used primary NK cells but with limited efficacy, pointing to the need to improve the functionality of these NK cells after their transfer to patients. The growing opportunities to augment NK cell functions have attracted several biotech companies to invest in NK cell research, spearheading NK therapy development with different innovative approaches. This review also stresses the need for combining adoptive transfer of allogeneic NK cells with NK function-augmenting products to achieve a maximum anti-tumor effect. As NK cells are safe to infuse, the use of CAR-NK cells may be instrumental in providing a much safer but still very effective platform, to bring CAR-based therapies to broader clinical applications. It may also facilitate effective tumor targeting of NK cells. oNKord^®^ and iPSC-derived NK cells could serve as alternative allogeneic platforms to develop CAR-NK products, besides NK cell lines. In a solid tumor setting, NK cells are challenged by several factors that affect their homing and penetration into the tumor tissues. Moreover, they should achieve and maintain an activated effector state, even in the face of immune suppressive conditions, that are prevalent in patients with cancer. To overcome these bottlenecks in NK therapy of solid tumors, a plethora of creative solutions are being pursued by numerous research labs as well as by biotech companies in clinical or close to clinical phase. Strategies to enhance NK cell functions from leading NK cell products are summarized in Figure 2. With all these exciting developments, NK cells are set to make a considerable impact on the future treatment of patients with hematological as well as with solid tumors.

## Author Contributions

JV, NK, HVV, TG, and JS wrote the paper. HVV, TG, HV, and JS reviewed the paper.

## Conflict of Interest Statement

JV, NK, and JS are employees of Glycostem Therapeutics. All authors declare they have no commercial, proprietary, or financial conflict of interest.

## References

[1] Luna JI Grossenbacher SK Murphy WJ Canter RJ. Targeting cancer stem cells with natural killer cell immunotherapy. Expert Opin Biol Ther (2017) 17(3):313–24. 10.1080/14712598.2017.1271874 27960589 PMC5311007

[2] Cooper MA Fehniger TA Caligiuri MA. The biology of human natural killer-cell subsets. Trends Immunol (2001) 22(11):633–40. 10.1016/S1471-4906(01)02060-9 11698225

[3] Campbell KS Hasegawa J. NK cell biology: an update and future directions. J Allergy Clin Immunol (2013) 132(3):536–44. 10.1016/j.jaci.2013.07.006 23906377 PMC3775709

[4] Zhang J Basher F Wu JD. NKG2D ligands in tumor immunity: two sides of a coin. Front Immunol (2015) 6:97. 10.3389/fimmu.2015.00097 25788898 PMC4349182

[5] de Andrade LF Smyth MJ Martinet L DNAM-1 control of natural killer cells functions through nectin and nectin-like proteins. Immunol Cell Biol (2014) 92(3):237–44. 10.1038/icb.2013.95 24343663

[6] Kruse PH Matta J Ugolini S Vivier E. Natural cytotoxicity receptors and their ligands. Immunol Cell Biol (2014) 92(3):221–9. 10.1038/icb.2013.98 24366519

[7] Borrego F Ulbrecht M Weiss EH Coligan JE Brooks AG. Recognition of human histocompatibility leukocyte antigen (HLA)-E complexed with HLA class I signal sequence-derived peptides by CD94/NKG2 confers protection from natural killer cell-mediated lysis. J Exp Med (1998) 187(5):813–8. 10.1084/jem.187.5.813 9480992 PMC2212178

[8] Pegram HJ Andrews DM Smyth MJ Darcy PK Kershaw MH. Activating and inhibitory receptors of natural killer cells. Immunol Cell Biol (2011) 89(2):216–24. 10.1038/icb.2010.78 20567250

[9] Leibson PJ. Signal transduction during natural killer cell activation: inside the mind of a killer. Immunity (1997) 6(6):655–61. 10.1016/S1074-7613(00)80441-0 9208838

[10] Zamai L Ahmad M Bennett IM Azzoni L Alnemri ES Perussia B. Natural killer (NK) cell-mediated cytotoxicity: differential use of TRAIL and Fas ligand by immature and mature primary human NK cells. J Exp Med (1998) 188(12):2375–80. 10.1084/jem.188.12.2375 9858524 PMC2212426

[11] Krasnova Y Putz EM Smyth MJ Souza-Fonseca-Guimaraes F. Bench to bedside: NK cells and control of metastasis. Clin Immunol (2015). 10.1016/j.clim.2015.10.001 26476139

[12] Ljunggren HG Karre K. In search of the ‘missing self’: MHC molecules and NK cell recognition. Immunol Today (1990) 11(7):237–44. 10.1016/0167-5699(90)90097-S 2201309

[13] Parham P Moffett A. Variable NK cell receptors and their MHC class I ligands in immunity, reproduction and human evolution. Nat Rev Immunol (2013) 13(2):133–44. 10.1038/nri3370 23334245 PMC3956658

[14] Rouas-Freiss N Moreau P Menier C LeMaoult J Carosella ED. Expression of tolerogenic HLA-G molecules in cancer prevents antitumor responses. Semin Cancer Biol (2007) 17(6):413–21. 10.1016/j.semcancer.2007.07.003 17881247

[15] Lee N Llano M Carretero M Ishitani A Navarro F Lopez-Botet M HLA-E is a major ligand for the natural killer inhibitory receptor CD94/NKG2A. Proc Natl Acad Sci U S A (1998) 95(9):5199–204. 10.1073/pnas.95.9.5199 9560253 PMC20238

[16] Schleypen JS Von Geldern M Weiss EH Kotzias N Rohrmann K Schendel DJ Renal cell carcinoma-infiltrating natural killer cells express differential repertoires of activating and inhibitory receptors and are inhibited by specific HLA class I allotypes. Int J Cancer (2003) 106(6):905–12. 10.1002/ijc.11321 12918068

[17] Jinushi M Takehara T Tatsumi T Hiramatsu N Sakamori R Yamaguchi S Impairment of natural killer cell and dendritic cell functions by the soluble form of MHC class I-related chain A in advanced human hepatocellular carcinomas. J Hepatol (2005) 43(6):1013–20. 10.1016/j.jhep.2005.05.026 16168521

[18] Hsia JY Chen JT Chen CY Hsu CP Miaw J Huang YS Prognostic significance of intratumoral natural killer cells in primary resected esophageal squamous cell carcinoma. Chang Gung Med J (2005) 28(5):335–40. 16086548

[19] Kondo E Koda K Takiguchi N Oda K Seike K Ishizuka M Preoperative natural killer cell activity as a prognostic factor for distant metastasis following surgery for colon cancer. Dig Surg (2003) 20(5):445–51. 10.1159/000072714 12900537

[20] Ishigami S Natsugoe S Tokuda K Nakajo A Che X Iwashige H Prognostic value of intratumoral natural killer cells in gastric carcinoma. Cancer (2000) 88(3):577–83. 10.1002/(SICI)1097-0142(20000201)88:3<577::AID-CNCR13>3.3.CO;2-M 10649250

[21] Villegas FR Coca S Villarrubia VG Jimenez R Chillon MJ Jareno J Prognostic significance of tumor infiltrating natural killer cells subset CD57 in patients with squamous cell lung cancer. Lung Cancer (2002) 35(1):23–8. 10.1016/S0169-5002(01)00292-6 11750709

[22] Burke S Lakshmikanth T Colucci F Carbone E. New views on natural killer cell-based immunotherapy for melanoma treatment. Trends Immunol (2010) 31(9):339–45. 10.1016/j.it.2010.06.003 20655806

[23] Ghiringhelli F Menard C Martin F Zitvogel L. The role of regulatory T cells in the control of natural killer cells: relevance during tumor progression. Immunol Rev (2006) 214:229–38. 10.1111/j.1600-065X.2006.00445.x 17100888

[24] Hoechst B Voigtlaender T Ormandy L Gamrekelashvili J Zhao F Wedemeyer H Myeloid derived suppressor cells inhibit natural killer cells in patients with hepatocellular carcinoma via the NKp30 receptor. Hepatology (2009) 50(3):799–807. 10.1002/hep.23054 19551844 PMC6357774

[25] Gabrilovich DI Ostrand-Rosenberg S Bronte V. Coordinated regulation of myeloid cells by tumours. Nat Rev Immunol (2012) 12(4):253–68. 10.1038/nri3175 22437938 PMC3587148

[26] Gill S Vasey AE De Souza A Baker J Smith AT Kohrt HE Rapid development of exhaustion and down-regulation of eomesodermin limit the antitumor activity of adoptively transferred murine natural killer cells. Blood (2012) 119(24):5758–68. 10.1182/blood-2012-03-415364 22544698 PMC3382935

[27] Schafer JL Müller-Trutwin MC Reeves RK. NK cell exhaustion: bad news for chronic disease? Oncotarget (2015) 6(26):21797–8. 10.18632/oncotarget.5490 26392410 PMC4673125

[28] Mamessier E Sylvain A Thibult ML Houvenaeghel G Jacquemier J Castellano R Human breast cancer cells enhance self tolerance by promoting evasion from NK cell antitumor immunity. J Clin Invest (2011) 121(9):3609–22. 10.1172/jci45816 21841316 PMC3171102

[29] Bauernhofer T Kuss I Henderson B Baum AS Whiteside TL. Preferential apoptosis of CD56dim natural killer cell subset in patients with cancer. Eur J Immunol (2003) 33(1):119–24. 10.1002/immu.200390014 12594840

[30] Parkhurst MR Riley JP Dudley ME Rosenberg SA. Adoptive transfer of autologous natural killer cells leads to high levels of circulating natural killer cells but does not mediate tumor regression. Clin Cancer Res (2011) 17(19):6287–97. 10.1158/1078-0432.CCR-11-1347 21844012 PMC3186830

[31] Rosenberg SA Lotze MT Muul LM Leitman S Chang AE Ettinghausen SE Observations on the systemic administration of autologous lymphokine-activated killer cells and recombinant interleukin-2 to patients with metastatic cancer. N Engl J Med (1985) 313(23):1485–92. 10.1056/NEJM198512053132327 3903508

[32] Rosenberg SA Restifo NP Yang JC Morgan RA Dudley ME. Adoptive cell transfer: a clinical path to effective cancer immunotherapy. Nat Rev Cancer (2008) 8(4):299–308. 10.1038/nrc2355 18354418 PMC2553205

[33] Geller MA Cooley S Judson PL Ghebre R Carson LF Argenta PA A phase II study of allogeneic natural killer cell therapy to treat patients with recurrent ovarian and breast cancer. Cytotherapy (2011) 13(1):98–107. 10.3109/14653249.2010.515582 20849361 PMC3760671

[34] Minculescu L Marquart HV Friis LS Petersen SL Schiødt I Ryder LP Early natural killer cell reconstitution predicts overall survival in T cell–replete allogeneic hematopoietic stem cell transplantation. Biol Blood Marrow Transplant (2016) 22(12):2187–93. 10.1016/j.bbmt.2016.09.006 27664326

[35] Wu CJ Ritz J. Induction of tumor immunity following allogeneic stem cell transplantation. Adv Immunol (2006) 90:133–73. 10.1016/S0065-2776(06)90004-2 16730263

[36] Ho VT Soiffer RJ. The history and future of T-cell depletion as graft-versus-host disease prophylaxis for allogeneic hematopoietic stem cell transplantation. Blood (2001) 98(12):3192–204. 10.1182/blood.V98.12.3192 11719354

[37] Antin JH T-cell depletion in GVHD: less is more? Blood (2011) 117(23):6061–2. 10.1182/blood-2011-04-348409 21659553

[38] Ruggeri L Capanni M Urbani E Perruccio K Shlomchik WD Tosti A Effectiveness of donor natural killer cell alloreactivity in mismatched hematopoietic transplants. Science (2002) 295(5562):2097–100. 10.1126/science.1068440 11896281

[39] Ruggeri L Capanni M Casucci M Volpi I Tosti A Perruccio K Role of natural killer cell alloreactivity in HLA-mismatched hematopoietic stem cell transplantation. Blood (1999) 94(1):333–9. 10381530

[40] Koepsell SA Miller JS McKenna DH Jr Natural killer cells: a review of manufacturing and clinical utility. Transfusion (2013) 53(2):404–10. 10.1111/j.1537-2995.2012.03724.x 22670662

[41] Spanholtz J Preijers F Tordoir M Trilsbeek C Paardekooper J de Witte T Clinical-grade generation of active NK cells from cord blood hematopoietic progenitor cells for immunotherapy using a closed-system culture process. PLoS One (2011) 6(6):e20740. 10.1371/journal.pone.0020740 21698239 PMC3116834

[42] Arai S Meagher R Swearingen M Myint H Rich E Martinson J Infusion of the allogeneic cell line NK-92 in patients with advanced renal cell cancer or melanoma: a phase I trial. Cytotherapy (2008) 10(6):625–32. 10.1080/14653240802301872 18836917

[43] Tonn T Schwabe D Klingemann HG Becker S Esser R Koehl U Treatment of patients with advanced cancer with the natural killer cell line NK-92. Cytotherapy (2013) 15(12):1563–70. 10.1016/j.jcyt.2013.06.017 24094496

[44] Passweg JR Tichelli A Meyer-Monard S Heim D Stern M Kuhne T Purified donor NK-lymphocyte infusion to consolidate engraftment after haploidentical stem cell transplantation. Leukemia (2004) 18(11):1835–8. 10.1038/sj.leu.2403524 15457184

[45] Koehl U Esser R Zimmermann S Tonn T Kotchetkov R Bartling T Ex vivo expansion of highly purified NK cells for immunotherapy after haploidentical stem cell transplantation in children. Klin Padiatr (2005) 217(6):345–50. 10.1055/s-2005-872520 16307421

[46] Shi J Tricot G Szmania S Rosen N Garg TK Malaviarachchi PA Infusion of haplo-identical killer immunoglobulin-like receptor ligand mismatched NK cells for relapsed myeloma in the setting of autologous stem cell transplantation. Br J Haematol (2008) 143(5):641–53. 10.1111/j.1365-2141.2008.07340.x 18950462 PMC3602915

[47] Yoon SR Lee YS Yang SH Ahn KH Lee J-H Lee J-H Generation of donor natural killer cells from CD34+ progenitor cells and subsequent infusion after HLA-mismatched allogeneic hematopoietic cell transplantation: a feasibility study. Bone Marrow Transplant (2010) 45(6):1038–46. 10.1038/bmt.2009.304 19881555

[48] Rizzieri DA Storms R Chen D-F Long G Yang Y Nikcevich DA Natural killer cell enriched donor lymphocyte infusions from a 3-6/6 HLA matched family member following non-myeloablative allogeneic stem cell transplantation. Biol Blood Marrow Transplant (2010) 16(8):1107–14. 10.1016/j.bbmt.2010.02.018 20188202 PMC3625653

[49] Brehm C Huenecke S Quaiser A Esser R Bremm M Kloess S IL-2 stimulated but not unstimulated NK cells induce selective disappearance of peripheral blood cells: concomitant results to a phase I/II study. PLoS One (2011) 6(11):e27351. 10.1371/journal.pone.0027351 22096557 PMC3212563

[50] Miller JS Soignier Y Panoskaltsis-Mortari A McNearney SA Yun GH Fautsch SK Successful adoptive transfer and in vivo expansion of human haploidentical NK cells in patients with cancer. Blood (2005) 105(8):3051–7. 10.1182/blood-2004-07-2974 15632206

[51] Copelan EA Hematopoietic Stem-Cell Transplantation. New Engl J Med (2006) 354(17):1813–26. 10.1056/NEJMra052638 16641398

[52] Stern M Passweg JR Meyer-Monard S Esser R Tonn T Soerensen J Pre-emptive immunotherapy with purified natural killer cells after haploidentical SCT: a prospective phase II study in two centers. Bone Marrow Transplant (2013) 48(3):433–8. 10.1038/bmt.2012.162 22941380

[53] Klingemann H Grodman C Cutler E Duque M Kadidlo D Klein AK Autologous stem cell transplant recipients tolerate haploidentical related-donor natural killer cell enriched infusions. Transfusion (2013) 53(2):412–8. 10.1111/j.1537-2995.2012.03764.x 22738379 PMC3549470

[54] Choi I Yoon SR Park SY Kim H Jung SJ Jang YJ Donor-derived natural killer cells infused after human leukocyte antigen-haploidentical hematopoietic cell transplantation: a dose-escalation study. Biol Blood Marrow Transplant (2014) 20(5):696–704. 10.1016/j.bbmt.2014.01.031 24525278

[55] Killig M Friedrichs B Meisig J Gentilini C Bluthgen N Loddenkemper C Tracking in vivo dynamics of NK cells transferred in patients undergoing stem cell transplantation. Eur J Immunol (2014) 44(9):2822–34. 10.1002/eji.201444586 24895051

[56] Shah NN Baird K Delbrook CP Fleisher TA Kohler ME Rampertaap S Acute GVHD in patients receiving IL-15/4-1BBL activated NK cells following T-cell–depleted stem cell transplantation. Blood (2015) 125(5):784–92. 10.1182/blood-2014-07-592881 25452614 PMC4311226

[57] Lee DA Denman CJ Rondon G Woodworth G Chen J Fisher T Haploidentical natural killer cells infused before allogeneic stem cell transplantation for myeloid malignancies: a phase I trial. Biol Blood Marrow Transplant (2016) 22(7):1290–8. 10.1016/j.bbmt.2016.04.009 27090958 PMC4905771

[58] Choi I Yoon SR Park SY Kim H Jung SJ Kang YL Donor-derived natural killer cell infusion after human leukocyte antigen-haploidentical hematopoietic cell transplantation in patients with refractory acute leukemia. Biol Blood Marrow Transplant (2016) 22(11):2065–76. 10.1016/j.bbmt.2016.08.008 27530969

[59] Shah N Li L McCarty J Kaur I Yvon E Shaim H Phase I study of cord blood-derived natural killer cells combined with autologous stem cell transplantation in multiple myeloma. Br J Haematol (2017) 177(3):457–66. 10.1111/bjh.14570 28295190 PMC5856008

[60] Iliopoulou EG Kountourakis P Karamouzis MV Doufexis D Ardavanis A Baxevanis CN A phase I trial of adoptive transfer of allogeneic natural killer cells in patients with advanced non-small cell lung cancer. Cancer Immunol Immunother (2010) 59(12):1781–9. 10.1007/s00262-010-0904-3 20703455 PMC11030924

[61] Rubnitz JE Inaba H Ribeiro RC Pounds S Rooney B Bell T NKAML: a pilot study to determine the safety and feasibility of haploidentical natural killer cell transplantation in childhood acute myeloid leukemia. J Clin Oncol (2010) 28(6):955–9. 10.1200/JCO.2009.24.4590 20085940 PMC2834435

[62] Bachanova V Burns LJ McKenna DH Curtsinger J Panoskaltsis-Mortari A Lindgren BR Allogeneic natural killer cells for refractory lymphoma. Cancer Immunol Immunother (2010) 59(11):1739–44. 10.1007/s00262-010-0896-z 20680271 PMC4082975

[63] Curti A Ruggeri L D’Addio A Bontadini A Dan E Motta MR Successful transfer of alloreactive haploidentical KIR ligand-mismatched natural killer cells after infusion in elderly high risk acute myeloid leukemia patients. Blood (2011) 118(12):3273–9. 10.1182/blood-2011-01-329508 21791425

[64] Bachanova V Cooley S Defor TE Verneris MR Zhang B McKenna DH Clearance of acute myeloid leukemia by haploidentical natural killer cells is improved using IL-2 diphtheria toxin fusion protein. Blood (2014) 123(25):3855–63. 10.1182/blood-2013-10-532531 24719405 PMC4064329

[65] Szmania S Lapteva N Garg T Greenway A Lingo J Nair B Ex vivo expanded natural killer cells demonstrate robust proliferation in vivo in high-risk relapsed multiple myeloma patients. J Immunother (2015) 38(1):24–36. 10.1097/CJI.0000000000000059 25415285 PMC4352951

[66] Kottaridis PD North J Tsirogianni M Marden C Samuel ER Jide-Banwo S Two-stage priming of allogeneic natural killer cells for the treatment of patients with acute myeloid leukemia: a phase I trial. PLoS One (2015) 10(6):e0123416. 10.1371/journal.pone.0123416 26062124 PMC4465629

[67] Rubnitz JE Inaba H Kang G Gan K Hartford C Triplett BM Natural killer cell therapy in children with relapsed leukemia. Pediatr Blood Cancer (2015) 62(8):1468–72. 10.1002/pbc.25555 25925135 PMC4634362

[68] Lim O Lee Y Chung H Her JH Kang SM Jung MY GMP-compliant, large-scale expanded allogeneic natural killer cells have potent cytolytic activity against cancer cells in vitro and in vivo. PLoS One (2013) 8(1):e53611. 10.1371/journal.pone.0053611 23326467 PMC3543306

[69] Yang Y Lim O Kim TM Ahn YO Choi H Chung H Phase I study of random healthy donor-derived allogeneic natural killer cell therapy in patients with malignant lymphoma or advanced solid tumors. Cancer Immunol Res (2016) 4(3):215–24. 10.1158/2326-6066.CIR-15-0118 26787822

[70] Shaffer BC Le Luduec J-B Forlenza C Jakubowski AA Perales M-A Young JW Phase II study of haploidentical natural killer cell infusion for treatment of relapsed or persistent myeloid malignancies following allogeneic hematopoietic cell transplantation. Biol Blood Marrow Transplant (2016) 22(4):705–9. 10.1016/j.bbmt.2015.12.028 26772158 PMC4801764

[71] Curti A Ruggeri L Parisi S Bontadini A Dan E Motta MR Larger size of donor alloreactive NK cell repertoire correlates with better response to NK cell immunotherapy in elderly acute myeloid leukemia patients. Clin Cancer Res (2016) 22(8):1914–21. 10.1158/1078-0432.CCR-15-1604 26787753

[72] Romee R Rosario M Berrien-Elliott MM Wagner JA Jewell BA Schappe T Cytokine-induced memory-like natural killer cells exhibit enhanced responses against myeloid leukemia. Sci Transl Med (2016) 8(357):ra123–357. 10.1126/scitranslmed.aaf2341 27655849 PMC5436500

[73] Dolstra H Roeven MW Spanholtz J Hangalapura BN Tordoir M Maas F Successful transfer of umbilical cord blood CD34+ hematopoietic stem and progenitor-derived NK cells in older acute myeloid leukemia patients. Clin Cancer Res (2017). 10.1158/1078-0432.ccr-16-2981 28280089

[74] Wang W Erbe AK Hank JA Morris ZS Sondel PM NK cell-mediated antibody-dependent cellular cytotoxicity in cancer immunotherapy. Front Immunol (2015) 6:368 10.3389/fimmu.2015.00368 26284063 PMC4515552

[75] Mellor J Brown M Irving H Zalcberg J Dobrovic A A critical review of the role of Fc gamma receptor polymorphisms in the response to monoclonal antibodies in cancer. J Hematol Oncol (2013) 6(1):1 10.1186/1756-8722-6-1 23286345 PMC3549734

[76] Ogura M Ishida T Hatake K Taniwaki M Ando K Tobinai K Multicenter phase II study of mogamulizumab (KW-0761), a defucosylated anti-CC chemokine receptor 4 antibody, in patients with relapsed peripheral T-cell lymphoma and cutaneous T-cell lymphoma. J Clin Oncol (2014) 32(11):1157–63. 10.1200/JCO.2013.52.0924 24616310

[77] Goede V Klein C Stilgenbauer S. Obinutuzumab (GA101) for the treatment of chronic lymphocytic leukemia and other B-cell non-hodgkin’s lymphomas: a glycoengineered type II CD20 antibody. Oncol Res Treat (2015) 38(4):185–92. 10.1159/000381524 25877943

[78] Cheney CM Stephens DM Mo X Rafiq S Butchar J Flynn JM Ocaratuzumab, an Fc-engineered antibody demonstrates enhanced antibody-dependent cell-mediated cytotoxicity in chronic lymphocytic leukemia. mAbs (2014) 6(3):749–55. 10.4161/mabs.28282 24594909 PMC4011919

[79] Oppenheim DE Spreafico R Etuk A Malone D Amofah E Peña-Murillo C Glyco-engineered anti-EGFR mAb elicits ADCC by NK cells from colorectal cancer patients irrespective of chemotherapy. Br J Cancer (2014) 110(5):1221–7. 10.1038/bjc.2014.35 24496456 PMC3950873

[80] Delord J-P Tabernero J García-Carbonero R Cervantes A Gomez-Roca C Bergé Y Open-label, multicentre expansion cohort to evaluate imgatuzumab in pre-treated patients with KRAS-mutant advanced colorectal carcinoma. Eur J Cancer (2014) 50(3):496–505. 10.1016/j.ejca.2013.10.015 24262587

[81] Ochoa MC Minute L Rodriguez I Garasa S Perez-Ruiz E Inoges S Antibody-dependent cell cytotoxicity (ADCC): immunotherapy strategies enhancing effector NK cells. Immunol Cell Biol (2017). 10.1038/icb.2017.6 28138156

[82] Kontermann RE Brinkmann U Bispecific antibodies. Drug Discov Today (2015) 20(7):838–47. 10.1016/j.drudis.2015.02.008 25728220

[83] Fan G Wang Z Hao M Li J Bispecific antibodies and their applications. J Hematol Oncol (2015) 8(1):130 10.1186/s13045-015-0227-0 26692321 PMC4687327

[84] Rothe A Sasse S Topp MS Eichenauer DA Hummel H Reiners KS A phase 1 study of the bispecific anti-CD30/CD16A antibody construct AFM13 in patients with relapsed or refractory Hodgkin lymphoma. Blood (2015) 125(26):4024–31. 10.1182/blood-2014-12-614636 25887777 PMC4528081

[85] Martin DW Williams SR Non-Natural MIC Proteins. Google Patents (2014).

[86] Sola C Chanuc F Thielens A Fuseri N Morel Y Bléry M Anti-tumoral efficacy of therapeutic human anti-KIR antibody (Lirilumab/BMS-986015/IPH2102) in a preclinical xenograft tumor model. J Immunother Cancer (2013) 1(Suppl 1):P40 10.1186/2051-1426-1-S1-P40

[87] Levy EM Bianchini M Von Euw EM Barrio MM Bravo AI Furman D Human leukocyte antigen-E protein is overexpressed in primary human colorectal cancer. Int J Oncol (2008) 32(3):633–41. 18292941

[88] Gooden M Lampen M Jordanova ES Leffers N Trimbos JB van der Burg SH HLA-E expression by gynecological cancers restrains tumor-infiltrating CD8(+) T lymphocytes. Proc Natl Acad Sci U S A (2011) 108(26):10656–61. 10.1073/pnas.1100354108 21670276 PMC3127933

[89] Glienke W Esser R Priesner C Suerth JD Schambach A Wels WS Advantages and applications of CAR-expressing natural killer cells. Front Pharmacol (2015) 6:21. 10.3389/fphar.2015.00021 25729364 PMC4325659

[90] Altvater B Landmeier S Pscherer S Temme J Schweer K Kailayangiri S 2B4 (CD244) signaling by recombinant antigen-specific chimeric receptors costimulates natural killer cell activation to leukemia and neuroblastoma cells. Clin Cancer Res (2009) 15(15):4857–66. 10.1158/1078-0432.CCR-08-2810 19638467 PMC2771629

[91] Zhang G Liu R Zhu X Wang L Ma J Han H Retargeting NK-92 for anti-melanoma activity by a TCR-like single-domain antibody. Immunol Cell Biol (2013) 91(10):615–24. 10.1038/icb.2013.45 24100387

[92] Sahm C Schonfeld K Wels WS. Expression of IL-15 in NK cells results in rapid enrichment and selective cytotoxicity of gene-modified effectors that carry a tumor-specific antigen receptor. Cancer Immunol Immunother (2012) 61(9):1451–61. 10.1007/s00262-012-1212-x 22310931 PMC11029748

[93] Chen X Han J Chu J Zhang L Zhang J Chen C A combinational therapy of EGFR-CAR NK cells and oncolytic herpes simplex virus 1 for breast cancer brain metastases. Oncotarget (2016) 7(19):27764–77. 10.18632/oncotarget.8526 27050072 PMC5053686

[94] Chu J Deng Y Benson DM He S Hughes T Zhang J CS1-specific chimeric antigen receptor (CAR)-engineered natural killer cells enhance in vitro and in vivo antitumor activity against human multiple myeloma. Leukemia (2014) 28(4):917–27. 10.1038/leu.2013.279 24067492 PMC3967004

[95] Jiang H Zhang W Shang P Zhang H Fu W Ye F Transfection of chimeric anti-CD138 gene enhances natural killer cell activation and killing of multiple myeloma cells. Mol Oncol (2014) 8(2):297–310. 10.1016/j.molonc.2013.12.001 24388357 PMC5528539

[96] Boissel L Betancur-Boissel M Lu W Krause DS Van Etten RA Wels WS Retargeting NK-92 cells by means of CD19- and CD20-specific chimeric antigen receptors compares favorably with antibody-dependent cellular cytotoxicity. Oncoimmunology (2013) 2(10):e26527. 10.4161/onci.26527 24404423 PMC3881109

[97] Klingemann H Boissel L Toneguzzo F. Natural killer cells for immunotherapy–advantages of the NK-92 cell line over blood NK cells. Front Immunol (2016) 7:91. 10.3389/fimmu.2016.00091 27014270 PMC4789404

[98] Adam J Planchard D Marabelle A Soria JC Scoazec JY Lantuejoul S. [PD-L1 expression: an emerging biomarker in non-small cell lung cancer]. Ann Pathol (2016) 36(1):94–102. 10.1016/j.annpat.2015.11.004 26778219

[99] Borcherding N Kusner D Liu G-H Zhang W. ROR1, an embryonic protein with an emerging role in cancer biology. Protein Cell (2014) 5(7):496–502. 10.1007/s13238-014-0059-7 24752542 PMC4085287

[100] Chang Y-H Connolly J Shimasaki N Mimura K Kono K Campana D. A chimeric receptor with NKG2D specificity enhances natural killer cell activation and killing of tumor cells. Cancer Res (2013) 73(6):1777–86. 10.1158/0008-5472.can-12-3558 23302231

[101] Kamiya T Chang YH Campana D. Expanded and activated natural killer cells for immunotherapy of hepatocellular carcinoma. Cancer Immunol Res (2016) 4(7):574–81. 10.1158/2326-6066.CIR-15-0229 27197065

[102] Torikai H Mi T Gragert L Maiers M Najjar A Ang S Genetic editing of HLA expression in hematopoietic stem cells to broaden their human application. Sci Rep (2016) 6:21757. 10.1038/srep21757 26902653 PMC4763194

[103] Carlsten M Childs RW. Genetic manipulation of NK cells for cancer immunotherapy: techniques and clinical implications. Front Immunol (2015) 6:266. 10.3389/fimmu.2015.00266 26113846 PMC4462109

[104] Torikai H Reik A Soldner F Warren EH Yuen C Zhou Y Toward eliminating HLA class I expression to generate universal cells from allogeneic donors. Blood (2013) 122(8):1341–9. 10.1182/blood-2013-03-478255 23741009 PMC3750336

[105] Delconte RB Kolesnik TB Dagley LF Rautela J Shi W Putz EM CIS is a potent checkpoint in NK cell-mediated tumor immunity. Nat Immunol (2016) 17(7):816–24. 10.1038/ni.3470 27213690

[106] Ng ES Davis RP Azzola L Stanley EG Elefanty AG. Forced aggregation of defined numbers of human embryonic stem cells into embryoid bodies fosters robust, reproducible hematopoietic differentiation. Blood (2005) 106(5):1601–3. 10.1182/blood-2005-03-0987 15914555

[107] Woll PS Martin CH Miller JS Kaufman DS. Human embryonic stem cell-derived NK cells acquire functional receptors and cytolytic activity. J Immunol (2005) 175(8):5095–103. 10.4049/jimmunol.175.8.5095 16210613

[108] Woll PS Grzywacz B Tian X Marcus RK Knorr DA Verneris MR Human embryonic stem cells differentiate into a homogeneous population of natural killer cells with potent in vivo antitumor activity. Blood (2009) 113(24):6094–101. 10.1182/blood-2008-06-165225 19365083 PMC2699231

[109] Knorr DA Ni Z Hermanson D Hexum MK Bendzick L Cooper LJ Clinical-scale derivation of natural killer cells from human pluripotent stem cells for cancer therapy. Stem Cells Transl Med (2013) 2(4):274–83. 10.5966/sctm.2012-0084 23515118 PMC3659832

[110] Shaim H Yvon E Cord blood: a promising source of allogeneic natural killer cells for immunotherapy. Cytotherapy (2015) 17(1):1–2. 10.1016/j.jcyt.2014.12.001 25527863

[111] Spanholtz J Tordoir M Eissens D Preijers F Meer A Joosten I High log-scale expansion of functional human natural killer cells from umbilical cord blood CD34-positive cells for adoptive cancer immunotherapy. PLoS One (2010) 5(2):e9221 10.1371/journal.pone.0009221 20169160 PMC2821405

[112] Cany J van der Waart AB Tordoir M Franssen GM Hangalapura BN de Vries J Natural killer cells generated from cord blood hematopoietic progenitor cells efficiently target bone marrow-residing human leukemia cells in NOD/SCID/IL2Rgnull mice. PLoS One (2013) 8(6):e64384 10.1371/journal.pone.0064384 23755121 PMC3673996

[113] Lehmann D Spanholtz J Osl M Tordoir M Lipnik K Bilban M Ex vivo generated natural killer cells acquire typical natural killer receptors and display a cytotoxic gene expression profile similar to peripheral blood natural killer cells. Stem Cells Dev (2012) 21(16):2926–38. 10.1089/scd.2011.0659 22571679 PMC3475144

[114] Veluchamy JP Heeren AM Spanholtz J van Eendenburg JD Heideman DA Kenter GG High-efficiency lysis of cervical cancer by allogeneic NK cells derived from umbilical cord progenitors is independent of HLA status. Cancer Immunol Immunother (2017) 66(1):51–61. 10.1007/s00262-016-1919-1 27783105 PMC5222919

[115] Veluchamy JP Lopez-Lastra S Spanholtz J Bohme F Kok N Heideman DAM In vivo efficacy of umbilical cord blood stem cell-derived NK cells in the treatment of metastatic colorectal cancer. Front Immunol (2017) 8:87. 10.3389/fimmu.2017.00087 28220124 PMC5292674

[116] Rosenberg SA. Interleukin-2 and the development of immunotherapy for the treatment of patients with cancer. Cancer J Sci Am (2000) 6(Suppl 1):S2–7. 10685650

[117] West WH Tauer KW Yannelli JR Marshall GD Orr DW Thurman GB Constant-infusion recombinant interleukin-2 in adoptive immunotherapy of advanced cancer. N Engl J Med (1987) 316(15):898–905. 10.1056/NEJM198704093161502 3493433

[118] Conlon KC Lugli E Welles HC Rosenberg SA Fojo AT Morris JC Redistribution, hyperproliferation, activation of natural killer cells and CD8 T cells, and cytokine production during first-in-human clinical trial of recombinant human interleukin-15 in patients with cancer. J Clin Oncol (2015) 33(1):74–82. 10.1200/JCO.2014.57.3329 25403209 PMC4268254

[119] Miller JS. Therapeutic applications: natural killer cells in the clinic. Hematology Am Soc Hematol Educ Program (2013) 2013:247–53. 10.1182/asheducation-2013.1.247 24319187

[120] Ito S Bollard CM Carlsten M Melenhorst JJ Biancotto A Wang E Ultra-low dose interleukin-2 promotes immune-modulating function of regulatory T cells and natural killer cells in healthy volunteers. Mol Ther (2014) 22(7):1388–95. 10.1038/mt.2014.50 24686272 PMC4089007

[121] Steel JC Waldmann TA Morris JC. Interleukin-15 biology and its therapeutic implications in cancer. Trends Pharmacol Sci (2012) 33(1):35–41. 10.1016/j.tips.2011.09.004 22032984 PMC3327885

[122] Imamura M Shook D Kamiya T Shimasaki N Chai SM Coustan-Smith E Autonomous growth and increased cytotoxicity of natural killer cells expressing membrane-bound interleukin-15. Blood (2014) 124(7):1081–8. 10.1182/blood-2014-02-556837 25006133

[123] Denman CJ Senyukov VV Somanchi SS Phatarpekar PV Kopp LM Johnson JL Membrane-bound IL-21 promotes sustained ex vivo proliferation of human natural killer cells. PLoS One (2012) 7(1):e30264. 10.1371/journal.pone.0030264 22279576 PMC3261192

[124] Oyer JL Pandey V Igarashi RY Somanchi SS Zakari A Solh M Natural killer cells stimulated with PM21 particles expand and biodistribute in vivo: clinical implications for cancer treatment. Cytotherapy (2016) 18(5):653–63. 10.1016/j.jcyt.2016.02.006 27059202

[125] Xu W Jones M Liu B Zhu X Johnson CB Edwards AC Efficacy and mechanism-of-action of a novel superagonist interleukin-15: interleukin-15 receptor α/Fc fusion complex in syngeneic murine models of multiple myeloma. Cancer Res (2013) 73(10):3075–86. 10.1158/0008-5472.CAN-12-2357 23644531 PMC3914673

[126] Rhode PR Egan JO Xu W Hong H Webb GM Chen X Comparison of the superagonist complex, ALT-803, to IL15 as cancer immunotherapeutics in animal models. Cancer Immunol Res (2016) 4(1):49–60. 10.1158/2326-6066.CIR-15-0093-T 26511282 PMC4703482

[127] Mathios D Park C-K Marcus WD Alter S Rhode PR Jeng EK Therapeutic administration of IL-15 superagonist complex ALT-803 leads to long-term survival and durable antitumor immune response in a murine glioblastoma model. Int J Cancer (2016) 138(1):187–94. 10.1002/ijc.29686 26174883 PMC4696021

[128] Gomes-Giacoia E Miyake M Goodison S Sriharan A Zhang G You L Intravesical ALT-803 and BCG treatment reduces tumor burden in a carcinogen induced bladder cancer rat model; a role for cytokine production and NK cell expansion. PLoS One (2014) 9(6):e96705. 10.1371/journal.pone.0096705 24896845 PMC4045574

[129] Kim PS Kwilas AR Xu W Alter S Jeng EK Wong HC IL-15 superagonist/IL-15RalphaSushi-Fc fusion complex (IL-15SA/IL-15RalphaSu-Fc; ALT-803) markedly enhances specific subpopulations of NK and memory CD8+ T cells, and mediates potent anti-tumor activity against murine breast and colon carcinomas. Oncotarget (2016) 7(13):16130–45. 10.18632/oncotarget.7470 26910920 PMC4941302

[130] Rosario M Liu B Kong L Collins LI Schneider SE Chen X The IL-15-based ALT-803 complex enhances FcgammaRIIIa-triggered NK cell responses and in vivo clearance of B cell lymphomas. Clin Cancer Res (2016) 22(3):596–608. 10.1158/1078-0432.CCR-15-1419 26423796 PMC4738096

[131] Fehniger TA Stuart RK Cooley SA Miller JS Curtsinger J Hillman TM Preliminary results of a phase 1/2 clinical trial of Cndo-109-activated allogeneic natural killer cells in high risk acute myelogenous leukemia patients in first complete remission. Blood (2014) 124(21):2320.25301333

[132] Krause SW Gastpar R Andreesen R Gross C Ullrich H Thonigs G Treatment of colon and lung cancer patients with ex vivo heat shock protein 70-peptide-activated, autologous natural killer cells: a clinical phase i trial. Clin Cancer Res (2004) 10(11):3699–707. 10.1158/1078-0432.CCR-03-0683 15173076

[133] Frei GM Persi N Lador C Peled A Cohen YC Nagler A Nicotinamide, a form of vitamin B3, promotes expansion of natural killer cells that display increased in vivo survival and cytotoxic activity. Blood (2011) 118(21):4035.

[134] Roccaro AM Mishima Y Sacco A Moschetta M Tai Y-T Shi J CXCR4 regulates extra-medullary myeloma through epithelial-mesenchymal transition-like transcriptional activation. Cell Rep (2015) 12(4):622–35. 10.1016/j.celrep.2015.06.059 26190113 PMC4961259

[135] Marasca R Maffei R NOX-A12: mobilizing CLL away from home. Blood (2014) 123(7):952–3. 10.1182/blood-2013-12-542480 24526776

[136] Chou T-C. Drug combination studies and their synergy quantification using the Chou-Talalay method. Cancer Res (2010) 70(2):440–6. 10.1158/0008-5472.can-09-1947 20068163

